# Supplementary material for: Effect of Zhan Zhuang Qigong on upper limb static tremor and aerobic exercise capacity in patients with mild-to-moderate Parkinson’s disease: study protocol for a randomised controlled trial
Source: BMJ Open. 2022 Jul 11;12(7):e059625. doi: 10.1136/bmjopen-2021-059625 (PMC9274526; doi:10.1136/bmjopen-2021-059625)
Supplement: Author's manuscript [file bmjopen-2021-059625.draft_revisions.pdf]

BMJ Open is committed to open peer review. As part of this commitment we make the peer review history of every article we publish publicly available.

When an article is published we post the peer reviewers' comments and the authors' responses online. We also post the versions of the paper that were used during peer review. These are the versions that the peer review comments apply to.

The versions of the paper that follow are the versions that were submitted during the peer review process. They are not the versions of record or the final published versions. They should not be cited or distributed as the published version of this manuscript.

BMJ Open is an open access journal and the full, final, typeset and author-corrected version of record of the manuscript is available on our site with no access controls, subscription charges or pay-per-view fees (http://bmjopen.bmj.com).

If you have any questions on BMJ Open's open peer review process please email info.bmjopen@bmj.com

BMJ Open

Effect of ZhanZhuang Qigong on upper limb static tremor and aerobic exercise capacity in patients with mild to moderate Parkinson's disease: Study Protocol for a Randomized controlled Trial

Journal:	*BMJ Open*
Manuscript ID	bmjopen-2021-059625
Article Type:	Protocol
Date Submitted by the Author:	29-Nov-2021

Complete List of Authors:	zhang, linlin; Shandong University of Traditional Chinese Medicine,<br>Liu, Xihua; Shandong University of Traditional Chinese Medicine Affiliated Hospital<br>Xi, Xiaoming; Shandong University of Traditional Chinese Medicine<br>Wang, Qing; Shandong University of Traditional Chinese Medicine<br>Qu, Xinjie; Shandong University of Traditional Chinese Medicine<br>Cao, Haihao; Shandong University of Traditional Chinese Medicine<br>Wang, Limin; Shandong University of Traditional Chinese Medicine<br>Sun, Wenyu; Shandong University of Traditional Chinese Medicine Affiliated Hospital<br>Chen, Guoming; Shandong University of Traditional Chinese Medicine Affiliated Hospital<br>Liu, Huifen; Shandong University of Traditional Chinese Medicine<br>Jiang, Xiaoyu; Shandong University of Traditional Chinese Medicine<br>Su, Hang; Shandong University of Traditional Chinese Medicine<br>Jiang, Jiahui; Shandong University of Traditional Chinese Medicine<br>Bi, Hongyan; Shandong University of Traditional Chinese Medicine Affiliated Hospital

Keywords:	Parkinson-s disease < NEUROLOGY, GERIATRIC MEDICINE, Rehabilitation medicine < INTERNAL MEDICINE, Clinical trials < THERAPEUTICS

SCHOLARONE™
Manuscripts

Effect of ZhanZhuang Qigong on upper limb static tremor and aerobic exercise capacity in patients with mild to moderate Parkinson's disease: Study Protocol for a Randomized controlled Trial

Linlin Zhang, MD[1];Xihua Liu,Ph.D[2];Xiaoming Xi,MD[1];Qing Wang,MD[1]; Xinjie Qu,MD[1];Haihao Cao[1],MD[1];Limin Wang[1], MD[1];Wenyu Sun,MD[2];Guoming Chen,MD[2];Huifen Liu,MD[1];Xiaoyu Jiang,MD[1]; Hang Su,MD[1];JiahuiJiang,MD[1]; Hongyan Bi,MD[1].

1: Shandong Traditional Chinese Medicine University,Jinan,China

2: Shandong Hospital of Traditional Chinese Medicine, Jinan,China

Correspondence

Hongyan Bi, Shandong Hospital of Traditional Chinese Medicine, Jinan,China

Tel:18800460276

Email:Hy__bi@163.com

For peer review only

Abstract

Introduction: At present, the first choice in the clinical treatment of Parkinson's disease static tremor is drug therapy with large side effects. In recent years, the effect of physical therapy on Parkinson's disease has become a research focus of Parkinson's disease. Some studies have proved that aerobic exercise and resistance exercise are beneficial to the alleviation of Parkinson's disease movement disorders and the improvement of aerobic exercise ability, but the effect of qigong on Parkinson's disease static tremor and aerobic exercise ability is still unknown.

Objective: To observe the effect of station post on upper limb static tremor and aerobic exercise capacity in patients with Parkinson's disease by rigorous randomized, parallel controlled, assignment hidden and evaluator blind method. **Methods**: Seventy-two patients with Parkinson's disease in Affiliated Hospital of Shandong University of Traditional Chinese Medicine were recruited and randomly divided into control group (n=36) and experimental group (n=36). The intervention group received conventional medicine plus station post qigong exercise for 8 weeks, 5 times a week, 30 minutes each time. The long-term effect of post qigong on Parkinson's disease was investigated at 8 weeks follow-up after intervention. Phyphox APP, CRST, CPET, UPDRS（Ⅱ、Ⅲ）, were used to evaluate tremor, aerobic exercise ability and motor function in Parkinson's disease patients. **Discussion**: This study will investigate the effect of ZhanZhuang qigong on upper limb static tremor and aerobic exercise capacity in Parkinson's disease. If the results are positive, it will add new research direction and evidence for the clinical exploration of exercise therapy for Parkinson's disease.

Strengths and limitations

1. At present, the clinical effect of physical therapy for Parkinson's tremor is not ideal, and there has been no clinical study on the effect of qigong on Parkinson's tremor.
2. The mobile phone tremor test and cardiopulmonary exercise test used in the outcome assessment have been clinically verified to be accurate and reliable.
3. This study will use a rigorous hidden allocation, randomized parallel controlled trial design that is more reliable and accurate than the pre - and post-trial controls previously used in Parkinson's clinical trials.
4. There may be staff turnover during the intervention period of the study, which may lead to

For peer review only - http://bmjopen.bmj.com/site/about/guidelines.xhtml

bias in the final results.

5. The study was unable to investigate forms of Parkinson's tremor other than static tremor.

Description: Parkinson's disease (PD) is a degenerative disease of the central nervous system common in middle-aged and elderly people[1]. According to statistics, the prevalence of PD in people aged 65-69 is about 0. 5-1%, and that in people aged 80 and above rises to 1-3%. With the aggravation of population aging, it is expected that the prevalence and incidence of PD will increase by more than 30% by 2030, which will bring tremendous influence and pressure to families and society. [2]

As one of the main motor symptoms of PD, static tremor is characterized by rhythmic and clay ball-like tremor with a large amplitude and frequency of 4-6Hz, which occurs more than when the weight of the affected limbs or other body structures is supported. It is aggravated when general exercise or pressure increases, and alleviates or disappears when the body moves at will. And gradually spread from the distal end of one limb to the other[3 4]. At present, the most effective method for clinical treatment of PD is still drug therapy[2 5 6]. Compound levodopa is considered to be the best treatment for the supplement of exogenous dopamine and the control of PD, but long-term use of Levodopa will gradually reduce the efficacy and lead to central and peripheral adverse reactions[7-9]. Moreover, levodopa has poor response to PD static tremor and has no effect on non-motor symptoms[10]. Invasive surgical treatments such as focused thalamotomy (FUS) and deep brain stimulation (DBS) provide significant relief of tremor in the short term, but do not prevent the progression of the neurodegenerative process of underlying PD[7 11-13]. In addition, surgery itself is risky and may be accompanied by complications[11].

In recent years, the improvement of PD symptoms by exercise therapy has attracted attention. A number of studies have shown that aerobic exercise and resistance exercise are beneficial to the alleviation of PD motor symptoms including tremor[6 14-19]. Among the traditional Chinese qigong therapies, Tai Chi, Wuqinxi and Baduanjin are also believed to enhance the balance ability of PD patients and correct abnormal gait[20-26]. However, there are no studies on PD tremor and aerobic capacity.

ZhanZhuang Qigong is the basic technique of Tai Chi, and it is a static, medium and low intensity aerobic exercise combining body and mind[27 28]. There have been a number of clinical studies on the benefits of Tai Chi on PD motor and non-motor functions. However, As Tai Chi is a highly difficult sport, most PD patients are middle-aged and elderly, and their learning ability is reduced. In addition, due to the limitations of venue and practice frequency, it takes at least 8 weeks to master it from the beginning to the preliminary stage, and even longer to master it well[29]. Shows a high time cost. The acceptability of patients decreased with the prolongation of time. As a basic skill of Tai Chi, the station post itself has many characteristics of Tai Chi. For example, the station post is also a movement combining body and mind, which controls the stability of limbs and trunk and the change of center of gravity with mind. In addition, like Tai Chi, ZhanZhuang is also a closed circular motion, and circular motion maintains the continuity and accuracy of action through adjustment, feedback, re-adjustment and re-feedback, which helps improve the body's ability to recognize stimuli and improve involuntary tremor[30 31].

For peer review only - http://bmjopen.bmj.com/site/about/guidelines.xhtml

Key words: ZhanZhuang qigong, Parkinson's disease, tremor, aerobic exercise capacity

Ethics and dissemination

This study has been approved by the Ethics Committee of Shandong University of Traditional Chinese Medicine (Approval Number: 2021-025-KY). The ETHICS Committee will be informed of any changes to the trial protocol, such as intervention intensity, outcome indicators and data collection The results of the study will be presented as a paper at an international conference or journal.

Test registration Number

This study protocol has been registered in the Chinese Clinical Trial Registry (Registration number: ChiCTR2100053529)

Version:

20211124

Method/Design

This study was a two-arm, randomized, parallel controlled trial with assignment hiding and evaluator blindness. The main objective is to investigate the effect of ZhanZhuang on upper limb static tremor and aerobic exercise ability in patients with mild to moderate Parkinson's disease, so as to provide a safe, effective, low-cost and easy-to-master aerobic exercise therapy for PD patients.

Patient and public involvement

The public is not involved in the design of the study. But we adjust the intensity of the intervention and other details based on how the participants respond to the intervention.

Materials and Methods

Study Design and Setting

The study will be conducted in the Affiliated Hospital of Shandong University of Traditional Chinese Medicine, and 72 patients diagnosed with PD with obvious upper limb static tremor will be included. Using random number table method, 72 patients were randomly divided into intervention group and control group in a 1:1 ratio. The basic information of the patient, such as gender, age, course of disease, education level, residence status and drug use, shall be registered and filled out. Tests for tremor and motor function were performed at least eight hours after patients stopped taking the drug.

The control group was treated with conventional medicine and routine aerobic exercise. According to the Chinese Guidelines for the Treatment of PD(4th edition), walking was selected as the aerobic exercise mode in the control group[32]. Five times a week, 30min each time, for 8 weeks. The combined cardiopulmonary exercise test (treadmill test) was used to measure peak oxygen uptake (peakVO_2) in Parkinson's patients at baseline and after intervention$_2$), anaerobic threshold (AT) and maximum heart rate (HRmax). Patients' exercise intensity reached HRmax (60%~80%) or self-induced fatigue Scale (RPE) grade 11~13. Changes in peak oxygen uptake (peakVO_2) and anaerobic threshold(AT) baseline and after intervention were evaluated for improvement in aerobic exercise capacity. The walking training was performed about 1 hour after the patient took the medication and the tremor symptoms resolved or disappeared.

The intervention group selected conventional medicine combined with ZhanZhuang

For peer review only - http://bmjopen.bmj.com/site/about/guidelines.xhtml

qigong. Invite an experienced qigong master to guide the ZhanZhuang. The first week was the learning phase, and the second to eighth week was the practice phase, and the practice was performed 5 times a week, 30min each time. The intensity reached HRmax (60%~80%) measured by cardiopulmonary combined exercise test or self-induced fatigue Scale (RPE) 11~13. Each patient was issued with a training instruction manual and a personal exercise calendar. The patient recorded the number of weekly exercises and the time of each exercise on the personal exercise calendar. The ZhanZhuang training was carried out about 1 hour after the patients took medication and the tremor symptoms were relieved or disappeared. One auxiliary staff will guide the safety.

Main points of qigong movement of ZhanZhuang:

Stand naturally, feet about 30 cm apart, feet parallel to the front; Arms around, hands naturally open, two fingertips about 15 cm apart; Knee flexion hip about 5~10 degrees; Hip forward(figures 1and 2).

Participants and Recruitment

With the Affiliated Hospital of Shandong University of Traditional Chinese Medicine as the platform, we visited and recruited the rehabilitation department and neurology department, and released recruitment advertisements through wechat.

To be assessed as having upper limb static tremor according to the Unified Parkinson's Rating Scale part III, and Hoehn-Yahr classification is in stages 1 to 3. (see Figure 3).

Inclusion criteria:

(1) Primary Parkinson's disease was in line with the recommended criteria of Chinese Clinical Diagnostic Criteria for Parkinson's Disease (2016);
(2) 50 to 70 years old;
(3) Improved Hoehn-Yahr stage 1 ~ 3;
(4) The condition is stable, take the necessary dose of Madopa, and keep standing during the "prescribed period";
(5) No serious cognitive dysfunction, can learn the ZhanZhuang movement; (MMSE score, illiteracy >17, primary school >20, middle school >22);
(6) Agree to participate in the study and group, and sign the informed consent.

Exclusion criteria:

(1) Secondary Parkinson's syndrome and Parkinson's superposition syndrome caused by cerebrovascular diseases, chemical drugs and other causes;
(2) Patients with serious heart, lung, liver, kidney dysfunction or cancer;
(3) The person cannot stand due to serious knee diseases such as severe knee osteoarthritis and knee trauma;
(4) Patients with dementia or serious mental diseases;
(5) Patients who are unable to stand or walk independently due to fracture, surgery or other reasons;
(6) no tremor;
(7) The "opening period" tremor is still obvious after taking the drug.

Termination/shedding criteria

(1) patients who do not cooperate with prescribed exercises or have poor compliance;
(2) Patients whose condition deteriorates or some complications occur during the course

For peer review only - http://bmjopen.bmj.com/site/about/guidelines.xhtml

of the study should not continue to accept the test.

Sample size

The study was designed as a randomized controlled trial and the main outcome was the mean relief of upper limb static tremor after treatment. A review of previous studies found that aerobic exercise can reduce the upper limb static tremor amplitude by about 60%. The average value of tremor was estimated to be (76±136) in the control group, and it was estimated to be reduced by 50 percent in the intervention group after training. Set the test level α as 0. 05 (bilateral), the degree of certainty 1-β as 0. 80, control group: intervention group =1:1. Calculated by *http://powerandsamplesize. com* need to 63 cases. Considering the abscission rate of about 15%, a total of 72 patients with Parkinson's disease should be included.

Randomization and Allocation Concealment and Blinding

This study was single-blind. Because of the particularity of qigong intervention, the training personnel and subjects are not blind. Blind test testers, data collectors, data statistics and analysis evaluators. Implementation of training, evaluation and statistical analysis of the separation of the way, that is, the training personnel do not contact the test personnel. Before the test, the intervention group and control group were changed to group A and group B. The test tester is only responsible for the test by number, and does not tell the group of the test subject. The data collection and processing personnel are separated, and the data collection personnel obtain the data of group A or group B according to the number and then hand it to the data statistician for statistical analysis. To ensure the objectivity of the results, the quality control personnel will break the blindness after the data statistics are completed.

The results of assessment

The primary outcome

(1) Tremor test

① Mobile phone movement test data collection

Phyphox APP[33]based on Android system(version: 1. 1. 9) collected the data of static tremor on the upper limb of patients with obvious tremor at a frequency of 100Hz and a time of 30 seconds.

Data collection of static tremor: The patient relaxed and sat in the chair, placed his hands on the armrest, and held the mobile phone with the screen up on the shaking side. At the same time, patients were asked to do numerical subtraction calculations to distract attention. Time from the beginning of the tremor and record a total of 30 seconds.

② The Clinical Tremor Rating Scale (CRST) A+B, CRST-A was used to evaluate the tremor at rest, postural, and intentional; Crst-b assesses handwriting (dominant hand only), wide and narrow spiral drawing, straight lines and casting. For the dominant hand, the maximum subscore of hand tremor was 32 for CRST(A+B) and 28 for non-dominant hand. The higher the score, the more severe the tremor.

(2) Combined Cardiopulmonary Exercise Test (CPET)

The cardiovit CS-200 cardiopulmonary combined exercise test system produced by SCHILLERA of Switzerland was used for CPET. The treadmill test was selected, and the increasing rate was set at 10w/min. According to the standards of harbor-UCLA Center cardiopulmonary Exercise Laboratory in the United States, the resting state lung function

For peer review only - http://bmjopen.bmj.com/site/about/guidelines.xhtml

examination, including fast lung capacity, slow lung capacity, maximum minute ventilatory capacity and diffusion function, should be completed first. Symptom limiting maximum CPET was then completed using continuous ramping regimen[34-36]. CPET is divided into four stages, including 3min of rest, 3min of warm-up (no power treadmill), and then exercise at a power increasing rate of 10W/min until symptom limiting maximum exercise, followed by more than 5min of recovery[37]. Continuous 12-lead ecg, non-invasive cuff blood pressure, and oxygen saturation measurements are required during the CPET process. Peak oxygen uptake was measured and calculated (peakVO)$_2$), anaerobic threshold (AT) and maximum heart rate (HR).

(3) Assessment of motor symptoms and daily living ability

(1) Unified Parkinson's Disease Rating Scale Part II and Part III (UPDRS II and III): The scale was divided into 4 parts. The first part is to evaluate the patient's mental behavior and emotion. Part II assessed the patient's ability to live daily, part III assessed the patient's motor function; The total score in part Ⅳ reflects patient complications. We selected partIIand part IIIto evaluate the daily living activities and motor functions of the patients respectively.

(2) Revised Hoehn-Yahr Grading Scale: The scale is divided into 8 stages from 0 to 5 (0, 1, 1. 5, 2, 2. 5, 3, 4 and 5), mainly for the evaluation of the severity of the disease. (table 1)

Adverse events

Adverse events that occurred during the study were recorded using a Case Report Form (CRF). For this trial, an adverse event will be defined as any adverse and unexpected signs, symptoms or diseases associated with the intervention, such as falls, joint injuries, hypertension, etc. Participants will be asked about their medical history and any adverse events they have experienced before the training begins, and adverse events will be asked and recorded after each intervention. The principal investigator will be notified within 24 hours if a serious adverse event (SAE) occurs. If subjects withdraw from the trial due to any adverse events, the study team will follow up according to protocol or until the adverse event is resolved, and record the follow-up information in the original file. The principal investigator manages safety reports. Any adverse events related to the intervention will be reported to the Ethics Review Committee of the Affiliated Hospital of Shandong University of Chinese Medicine.

Statistical analysis

The collected basic patient information, mobile phone exercise test data, CRST score, UPDRS(II,III) score were exported to the computer in CSV format.

SPSS17.0 statistical software was used for data processing and analysis: 1. Measurement data were measured by mean standard deviation (x^2±s); 2. T test: normal distribution and homogeneity of the overall variance of the two samples; 3. T test: normal distribution but uneven overall variance; 4. Wilcoxon rank sum test: it does not conform to normal distribution; The test level of statistical inference P<0.05 indicates statistical

For peer review only - http://bmjopen.bmj.com/site/about/guidelines.xhtml

significance.

Mobile exercise test data

The original form is the acceleration a(m/s) of three axes (X, Y and Z axes) vector direction respectively), rotation radian w (rad/s). MATLAB(version R 2018a, MathWorks Inc, USA) computer programming program was used to perform Fourier transform on the data, and the acceleration and rotation radian measured by smart phone were converted to the corresponding frequency, peak amplitude and speed. The final phone test data will be presented as the amplitude and frequency of the static tremor. Then normal distribution test was conducted. Since the sample size of this study was small (n<5000), Shapiro-Wilk normal test (S-W test) was used. P>0.05 belonged to normal distribution, while P<0.05 belonged to non-normal distribution.

According to the results of the normal distribution test, if the mobile phone exercise test data met the normal distribution and the variance was uniform, then the mobile phone exercise test data of PD patients in different groups were tested with two samples independent T test. P<0.05 indicated that the exercise test had statistically significant difference between groups: If the data did not meet the normal distribution, Non-parametric test (median test and Mann-Whitney U test) was performed for comparison. P<0.05 in median test indicated that the difference in central tendency of this score between groups was statistically significant.

CPET test data

After the subjects completed the CPET test, the raw data from the subjects' testing process will be saved. According to the standardized principles of CPET data analysis and interpretation of FDA clinical trial in Harbor-UCLA Medical Center[38 39], the breath-by-breath original data of the subjects were segmsed every second from the CPET system software. Then average the data for 10s and export the average data[36 40]. The warm-up value is the average of the last 30s of the warm-up period, the anaerobic threshold value is the data value of 10s when the anaerobic threshold, and the peak value is the average of 30s during the extreme exercise. The method to determine the anaerobic threshold is v-Slope method, that is, the mean value of 10s of oxygen uptake when the Co_2 emission rate is significantly accelerated relative to the oxygen uptake rate in the process of movement[41].

Discussion

PD not only reduces the quality of life of patients, but also brings heavy economic burden to society and families. Upper limb static tremor is one of the main motor symptoms of Parkinson's disease. Dyskinesia can also lead to decreased activity and aerobic exercise ability in PD patients. At present, non-drug therapy for Parkinson's disease mainly includes aerobic exercise and resistance training. Previous studies have found that aerobic exercise and resistance training are beneficial to tremor, bradykinesia, balance ability and quality of life of Parkinson's patients[42]. However, the effect of qigong on PD static tremor and aerobic ability is still unknown.

Free radical(OH)[43] is a strong oxide that can cause lipid peroxidation of unsaturated fatty acids and produce lipid peroxidation (LPO), which damages proteins and DNA and leads to cell degeneration and death Oxidative Stress (OS)[44] refers to a state of imbalance between oxidation and antioxidant effects in the body and tendency to

For peer review only - http://bmjopen.bmj.com/site/about/guidelines.xhtml

oxidation, which is considered to be an important factor leading to aging and disease. Due to the increased activity of B-monoamine oxidase (MAO-B) in Parkinson's patients, excessive free radicals can be produced to oxidize and destroy cell membranes[45]. At the same time of oxidation, dopamine oxidation products in substantia nigra cells polymerize to form neuromelanin, which combines with iron to produce Fenton reaction and form free radicals. Under normal circumstances, there are enough antioxidant substances in cells, such as glutathione (GSH), glutathione peroxidase (GSH-PX) and superoxide dismutase (SOD) in the brain, and free radicals generated by dopamine oxidation will not lead to oxidative stress[46 47]. In Parkinson's disease, the substantia nigra is vulnerable to oxidative stress due to decreased glutathione, increased lipid peroxide, increased iron ion concentration and decreased ferritin content[46-48]. Studies have found that[10 49] ZhanZhuang can improve the activity level of superoxide dismutase (SOD) in human body, prevent the harm of superoxide free radical to human body, and play a positive role in fighting aging, inflammation and immune diseasesTherefore, the station post may protect the substantia nigra of Parkinson's patients by increasing the activity level of Superoxide dismutase and reduce the lesion of substantia nigra, thus slowing down the progression of Parkinson's disease and improving the symptoms of Parkinson's patients.

At present, the generally accepted mechanism of PD tremor in clinical practice is the induced-switch-regulation model proposed by Duval[4] : Induced: Pathological activity of basal nucleus striatum induces the rhythmic cluster discharge activity of thalamic neurons through excessive input of inhibitory signals to thalamic cells and change of discharge mode. Switch: the oscillations of the internal and external network of thalamus combine with each other to make the oscillations continuously integrate and strengthen until they reach the threshold value of producing tremor. Regulation: After PD tremor-related oscillations are introduced into the cerebellum, the cerebellum processes them as random movements and adjusts the tremors to ensure the stability of oscillation activities such as frequency and amplitude. The tremors transmitted to the cerebral cortex are then transmitted to the periphery via the cortical - spinal cord pathway in the form of oscillations, thus being reflected as the tremors of the body. Yang[15], based on the data from the basal ganglia injury model in mice and rats, found that exercise training can reduce the overactivation of the basal ganglia cortical circuit, thus weakening the transmission of tremor signals, and thus alleviating tremor. Recent studies have found that this animal model is also applicable to humans[16]. Mona[50]found that upper limb centrifugal exercise can effectively reduce upper limb tremor amplitude in Parkinson's disease patientsStanding piling as an aerobic exercise may also reduce tremors at the motor level.

PD tremor is influenced by attention and stress levels[51]. Studies have found that tremor is intentionally attenuated when attention is focused on it, and PD tremor is amplified by stress[52]. Jiaqiu Wang[53] using magnetic resonance (NMR) to tremor subtypes in patients with Parkinson's disease data collection and analysis, found that the brains of tremor subtypes in patients with a wide range of functional connection between the frontal lobe and cerebellum, and UPDRS score Ⅲ movement and the cognitive function score also existed significant correlation, that movement function and cognitive function in patients with PD has a high degree of correlation. DA substitution therapy can

For peer review only - http://bmjopen.bmj.com/site/about/guidelines.xhtml

alleviate some motor characteristics of PD, but the observed beneficial effect on cognitive function is small[54]. A number of studies[27 55 56] have shown that ZhanZhuangs can enhance the attention and cognitive ability of adolescents and the elderly. Yiyue Chen[57] found that ZhanZhuang exercise would lead to an increase in the amplitude of P3 wave in frontal region, central region and top region, with a shorter incubation period than baseline. The amplitude of P3 wave reflects the allocation of neural electrical resources, and the latency is related to the speed of stimulus classification. These results suggest that station post can affect the neural electrical processing related to cognitive control by changing neural electrical resource allocation and stimulus classification speed, thus affecting cognitive function. Therefore, the station post may indirectly improve the tremor performance by enhancing the cognitive ability of PD patients.

Executive control is a general control mechanism in which individuals coordinate various cognitive processes and resources to ensure that the cognitive system implements specific goals in a flexible and optimized way when completing complex cognitive tasks. Executive control focuses on the coordination of brain commands and muscle movements. Sihuan Yang[58] conducted a one-year ZhanZhuang exercise for healthy college students, and found that the eeg activity coefficient of those students who participated in the ZhanZhuang exercise generally decreased, and the EEG showed a high trend of synchronization. The degree of ordering of the whole brain was improved, and the brain activities were more coordinated and synchronized. ChaoFang[59] found in their study that ZhanZhuangs can exercise the muscles of the whole body statically, provide benign stimulation to the brain and adjust the disorder of excitement and inhibition of the cerebral cortex. The study of Jianguo Ma[60] also confirmed that ZhanZhuangs can improve the response speed and accuracy of subjects, and improve the executive control level of the brain. ZhanZhuang is an aerobic exercise combining the body and mind, focusing on controlling the body with the mind and mobilizing the muscle groups of the trunk and limbs to participate in it[27].This combination of body and mind can stimulate the nervous system and increase its sensitivity, so that the more efficient and precise control of muscle work. In addition, ZhanZhuangs put the body in a stress state of postural control for a long time, and clinical studies believe that stress can significantly affect neural plasticity[60]. Higher levels of executive control and finer, more efficient postural control may be beneficial for Parkinson's disease tremor relief.

YU Guo[27] found that B cell receptor signaling pathway and NF-κB signaling pathway were significantly enriched in college students after ZhanZhuang, suggesting that ZhanZhuang may enhance immunity and improve inflammatory microenvironment in vivo by activating B cell receptor signaling pathway and improving T lymphocyte level, or regulating NF-κB signaling pathway. Regulate the metabolism of valine, leucine and isoleucine, prevent the excessive accumulation of glutamate, increase the content of astrocytes and 5-hydroxytryptamine, and prevent the necrosis and apoptosis of neurons. Specific regulation of lysosomal pathway mediates the level of autophagy, improves the survival level of neurons, and thus alleviates depression and improves adverse psychiatric symptoms. A number of studies[27 60-65] have also shown that station post can significantly improve blood flow, improve inflammatory environment, and may enhance body immunity.

For peer review only - http://bmjopen.bmj.com/site/about/guidelines.xhtml

Footnotes

The author contributions

All authors contributed to the design of the study. HYB and XHL provided the research idea, LLZ and XMX designed the experiment, QW, XJQ, HHC, LMW for Chaired the ethics application, and GMC and WYS contributed to the data analysis project. JHJ, HS, XYJ and HFL drafted the manuscript.All authors approved the final version of the manuscript.

Funding

This study was funded by the Science and Technology Project of Traditional Chinese Medicine of Shandong Province sponsored by Shandong Provincial Health Commission (Grant Number: 2020Z04). However, the sponsor is not involved in the conduct of the trial and the collection and statistics of the data. The final results of the trial and the publication of the report are independent of the sponsor.

Consent for publication

Don't need.

Competing interests

The authors declare that they have no competing interests.

Table 1

Schedule for data collection, the process of the assessments per visits.

Measures	Baseline week	Intervention period (0-8 weeks)	End of intervention (9 weeks)	Follow-up period (9-16 weeks)	Follow-up (17 weeks)
Participants' characteristics	√				
Mobile phone tremor test	√		√		√
CRST(A+B)	√		√		√
CPET	√		√		√
UPDRS(Ⅱ Ⅲ)	√		√		√
Hoehn-Yahr	√		√		√
Adverse experiences[1]		√		√	
Combined medication[2]		√		√	

[1]***Adverse experiences*** *: Any adverse experiences at arry visit during treatment sessions and 36 weeks will be monitored. The research team will review all trial protocols, monitor patient safety, and imvestigate arry adverse events.*

[2]***Combined medication:*** *Patients will be asked whether they have used other medications during the treatment. If they have usedconcomitant medications, then the type and dose ofmedication taken by them will also be recorded in detail. CRST(A+B):Clinical tremor Rating Scale; CPET:Cardiopulmonary exercise test; UPDRS(Ⅱ Ⅲ):Unified Parkinson's Disease Rating Scale Ⅱ III.*

Figure legend

Figure 1 and 2: Demonstration of qigong movement of ZhangZhuang

Figure 3: Test flow chart

Table 1:Schedule for data collection, the process of the assessments per visits.

For peer review only - http://bmjopen.bmj.com/site/about/guidelines.xhtml

References

1. Cryan JF, O'Riordan KJ, Sandhu K. The gut microbiome in neurological disorders. *The Lancet Neurology* 2020;19(2):179-94. doi: 10.1016/s1474-4422(19)30356-4 [published Online First: 2019/11/23]

2. Hayes MT. Parkinson's Disease and Parkinsonism. *The American journal of medicine* 2019;132(7):802-07. doi: 10.1016/j.amjmed.2019.03.001 [published Online First: 2019/03/21]

3. Meshack RP, Norman KE. A randomized controlled trial of the effects of weights on amplitude and frequency of postural hand tremor in people with Parkinson's disease. *Clinical rehabilitation* 2002;16(5):481-92. doi: 10.1191/0269215502cr521oa [published Online First: 2002/08/27]

4. Bian YJ, Wang YX, Zhou YQ. Research progress on brain network model of tremor mechanism in Parkinson's disease. *Clinical Medicine of China* 32 (8) : 749-53. Doi: 10.3760 / cma.i SSN. 1008-6315.2016.08.023

5. Sarno M, Gaztanaga W, Banerjee N. Revisiting eligibility for deep brain stimulation: Do preoperative mood symptoms predict outcomes in Parkinson's disease patients? *Parkinsonism & related disorders* 2019;63:131-36. doi: 10.1016/j.parkreldis.2019.02.019 [published Online First: 2019/02/26]

6. Armstrong MJ, Okun MS. Choosing a Parkinson Disease Treatment. *Jama* 2020;323(14):1420. doi: 10.1001/jama.2020.1224 [published Online First: 2020/04/15]

For peer review only - http://bmjopen.bmj.com/site/about/guidelines.xhtml

7. Cerri S, Blandini F. An update on the use of non-ergot dopamine agonists for the treatment of Parkinson's disease. *Expert opinion on pharmacotherapy* 2020;21(18):2279-91. doi: 10.1080/14656566.2020.1805432 [published Online First: 2020/08/18]

8. Liu HJ, He TZ, Zhang JL. [Systematic review and screening of basic Chinese herbs for traditional Chinese medicine compounds combined with levodopa medicine in treatment of Parkinson's disease]. *Zhongguo Zhong yao za zhi = Zhongguo zhongyao zazhi = China journal of Chinese materia medica* 2020;45(24):6043-52. doi: 10.19540/j.cnki.cjcmm.20200717.501 [published Online First: 2021/01/27]

9. McGill A, Houston S, Lee RY. Dance for Parkinson's: a new framework for research on its physical, mental, emotional, and social benefits. *Complementary therapies in medicine* 2014;22(3):426-32. doi: 10.1016/j.ctim.2014.03.005 [published Online First: 2014/06/08]

10. Bond AE, Shah BB, Huss DS. Safety and Efficacy of Focused Ultrasound Thalamotomy for Patients With Medication-Refractory, Tremor-Dominant Parkinson Disease: A Randomized Clinical Trial. *JAMA Neurol* 2017;74(12):1412-18. doi: 10.1001/jamaneurol.2017.3098 [published Online First: 2017/10/31]

11. Giordano M, Caccavella VM, Zaed I. Comparison between deep brain stimulation and magnetic resonance-guided focused ultrasound in the treatment of essential tremor: a systematic review and pooled analysis of functional outcomes. *Journal of neurology, neurosurgery, and psychiatry* 2020;91(12):1270-78. doi: 10.1136/jnnp-2020-323216 [published Online First: 2020/10/16]

12. Kremer NI, Pauwels RWJ, Pozzi NG. Deep Brain Stimulation for Tremor: Update on Long-

For peer review only

For peer review only - http://bmjopen.bmj.com/site/about/guidelines.xhtml

Term Outcomes, Target Considerations and Future Directions. *Journal of clinical medicine* 2021;10(16) doi: 10.3390/jcm10163468 [published Online First: 2021/08/28]

13. Pooja NA, Pahuja SK, Veer K. Significance of MRI Guided Focused Ultrasound Thalamotomy for Parkinson's Disease: A Review. *Current medical imaging* 2021;17(6):714-19. doi: 10.2174/1573405616666201223142505 [published Online First: 2020/12/29]

14. Petzinger GM, Fisher BE, Van Leeuwen JE. Enhancing neuroplasticity in the basal ganglia: the role of exercise in Parkinson's disease. *Movement disorders : official journal of the Movement Disorder Society* 2010;25 Suppl 1(0 1):S141-5. doi: 10.1002/mds.22782 [published Online First: 2010/02/27]

15. Yang J, Sadler TR, Givrad TK. Changes in brain functional activation during resting and locomotor states after unilateral nigrostriatal damage in rats. *NeuroImage* 2007;36(3):755-73. doi: 10.1016/j.neuroimage.2007.03.010 [published Online First: 2007/05/08]

16. Petzinger GM, Fisher BE, McEwen S. Exercise-enhanced neuroplasticity targeting motor and cognitive circuitry in Parkinson's disease. *The Lancet Neurology* 2013;12(7):716-26. doi: 10.1016/s1474-4422(13)70123-6 [published Online First: 2013/06/19]

17. Tomlinson CL, Herd CP, Clarke CE. Physiotherapy for Parkinson's disease: a comparison of techniques. *The Cochrane database of systematic reviews* 2014;2014(6):Cd002815. doi: 10.1002/14651858.CD002815.pub2 [published Online First: 2014/06/18]

18. de Oliveira Gondim ITG, de Souza CCB, Rodrigues MAB. Portable accelerometers for the

For peer review only - http://bmjopen.bmj.com/site/about/guidelines.xhtml

evaluation of spatio-temporal gait parameters in people with Parkinson's disease: an integrative review. *Archives of gerontology and geriatrics* 2020;90:104097. doi: 10.1016/j.archger.2020.104097 [published Online First: 2020/06/13]

19. de Oliveira MPB, Lobato DFM, Smaili SM. Effect of aerobic exercise on functional capacity and quality of life in individuals with Parkinson's disease: A systematic review of randomized controlled trials. *Archives of gerontology and geriatrics* 2021;95:104422. doi: 10.1016/j.archger.2021.104422 [published Online First: 2021/05/02]

20. Amano S, Nocera JR, Vallabhajosula S. The effect of Tai Chi exercise on gait initiation and gait performance in persons with Parkinson's disease. *Parkinsonism & related disorders* 2013;19(11):955-60. doi: 10.1016/j.parkreldis.2013.06.007 [published Online First: 2013/07/10]

21. Chen S, Zhang Y, Wang YT. The effect of Qigong-based therapy on patients with Parkinson's disease: a systematic review and meta-analysis. *Clinical rehabilitation* 2020;34(12):1436-48. doi: 10.1177/0269215520946695 [published Online First: 2020/07/31]

22. Deuel LM, Seeberger LC. Complementary Therapies in Parkinson Disease: a Review of Acupuncture, Tai Chi, Qi Gong, Yoga, and Cannabis. *Neurotherapeutics : the journal of the American Society for Experimental NeuroTherapeutics* 2020;17(4):1434-55. doi: 10.1007/s13311-020-00900-y [published Online First: 2020/08/14]

23. Fidan O, Seyyar GK, Aras B. The effect of Tai Chi and Qigong on health-related quality of life in Parkinson's disease: a systematic review and meta-analysis of systematic

For peer review only - http://bmjopen.bmj.com/site/about/guidelines.xhtml

reviews. *International journal of rehabilitation research Internationale Zeitschrift fur Rehabilitationsforschung Revue internationale de recherches de readaptation* 2019;42(3):196-204. doi: 10.1097/mrr.0000000000000358 [published Online First: 2019/05/23]

24. Jin X, Wang L, Liu S. The Impact of Mind-body Exercises on Motor Function, Depressive Symptoms, and Quality of Life in Parkinson's Disease: A Systematic Review and Meta-analysis. *International journal of environmental research and public health* 2019;17(1) doi: 10.3390/ijerph17010031 [published Online First: 2019/12/22]

25. Song R, Grabowska W, Park M. The impact of Tai Chi and Qigong mind-body exercises on motor and non-motor function and quality of life in Parkinson's disease: A systematic review and meta-analysis. *Parkinsonism & related disorders* 2017;41:3-13. doi: 10.1016/j.parkreldis.2017.05.019 [published Online First: 2017/06/13]

26. Yang Y, Hao YL, Tian WJ. The effectiveness of Tai Chi for patients with Parkinson's disease: study protocol for a randomized controlled trial. *Trials* 2015;16:111. doi: 10.1186/s13063-015-0639-8 [published Online First: 2015/04/16]

27. Guo Yu. Study on physiological and gene expression mechanism of "Regulating heart" effect of three-circle station post on alleviating depression [PhD]. Beijing University of Chinese Medicine, 2019.

28. Gao Yongmei. Research on the Special Role and Value of Taiji Post in Modern Wushu. *Martial Arts Research* . 2016;1(03):81-83.

29. Li F, Harmer P, Fitzgerald K. Tai chi and postural stability in patients with Parkinson's disease. *The New England journal of medicine* 2012;366(6):511-9. doi:

For peer review only - http://bmjopen.bmj.com/site/about/guidelines.xhtml

10.1056/NEJMoa1107911 [published Online First: 2012/02/10]

30. Zhang Y Z, Ma Z, Zheng M Y. Effect of Taijiquan on balance disorder in elderly Parkinson's disease and rheumatism related diseases. *Chinese Journal of Gerontology*. 2016; 36 (23) : 6031-33.

31. Zhang L, Jiang W F, Sun C H. Progress of Tai Chi exercise in the treatment of motor and non-motor symptoms of Parkinson's disease. Journal of Neurology and Neurorehabilitation. 2017; 13 (3) : 138-41.

32. Parkinson's disease and Movement Disorders Group, Neurology Branch of Chinese Medical Association, Parkinson's disease and Movement Disorders Group, Neurology Branch of Chinese Medical Doctor Association. Chinese guidelines for the treatment of Parkinson's disease (4th edition). Chinese Journal of Neurology. 2020; 53 (12) : 973-86.

33. Xu MENGyuan. Quantitative analysis and comparison of UPDRS and smart phones on upper limb tremor in PD patients [Master's degree]. Kunming Medical University, 2020.

34. ATS/ACCP Statement on cardiopulmonary exercise testing. *American journal of respiratory and critical care medicine* 2003;167(2):211-77. doi: 10.1164/rccm.167.2.211 [published Online First: 2003/01/14]

35. Balady GJ, Arena R, Sietsema K. Clinician's Guide to cardiopulmonary exercise testing in adults: a scientific statement from the American Heart Association. *Circulation* 2010;122(2):191-225. doi: 10.1161/CIR.0b013e3181e52e69 [published Online First: 2010/06/30]

For peer review only - http://bmjopen.bmj.com/site/about/guidelines.xhtml

36. Sun Xing-guo. standardized operation requirements and difficulties of cardiopulmonary exercise test - data analysis diagram and interpretation principles. Chinese Journal of Applied Physiology. 2015; 31 (4) : 361-65 + 96-98.

37. Song YA. Clinical study of radial pulse wave in cardiopulmonary exercise test as the core of the overall management of chronic disease [Ms]. Chongqing Medical University, 2021.

38. Hansen JE, Sun XG, Stringer WW. A simple new visualization of exercise data discloses pathophysiology and severity of heart failure. *Journal of the American Heart Association* 2012;1(3):e001883. doi: 10.1161/jaha.112.001883 [published Online First: 2012/11/07]

39. Sun XG, Hansen JE, Beshai JF. Oscillatory breathing and exercise gas exchange abnormalities prognosticate early mortality and morbidity in heart failure. *Journal of the American College of Cardiology* 2010;55(17):1814-23. doi: 10.1016/j.jacc.2009.10.075 [published Online First: 2010/04/24]

40. Sun Xinguo. New 9 images of cardiopulmonary exercise test to enhance cardiopulmonary metabolism and other overall functions .*Chinese Journal of Applied Physiology.* 2015; 31 (4) : 369-73 + 99-400.

41. Beaver WL, Wasserman K, Whipp BJ. A new method for detecting anaerobic threshold by gas exchange. *Journal of applied physiology (Bethesda, Md : 1985)* 1986;60(6):2020-7. doi: 10.1152/jappl.1986.60.6.2020 [published Online First: 1986/06/01]

42. Kim Y, Lai B, Mehta T. Exercise Training Guidelines for Multiple Sclerosis, Stroke, and Parkinson Disease: Rapid Review and Synthesis. *American journal of physical*

For peer review only - http://bmjopen.bmj.com/site/about/guidelines.xhtml

medicine & rehabilitation 2019;98(7):613-21. doi: 10.1097/phm.0000000000001174 [published Online First: 2019/03/08]

43. Sun MS, Jin H, Sun X. Free Radical Damage in Ischemia-Reperfusion Injury: An Obstacle in Acute Ischemic Stroke after Revascularization Therapy. *Oxidative medicine and cellular longevity* 2018;2018:3804979. doi: 10.1155/2018/3804979 [published Online First: 2018/05/18]

44. Chen Z, Zhong C. Oxidative stress in Alzheimer's disease. *Neuroscience bulletin* 2014;30(2):271-81. doi: 10.1007/s12264-013-1423-y [published Online First: 2014/03/26]

45. Oertel W, Schulz JB. Current and experimental treatments of Parkinson disease: A guide for neuroscientists. *Journal of neurochemistry* 2016;139 Suppl 1:325-37. doi: 10.1111/jnc.13750 [published Online First: 2016/09/01]

46. Chang KH, Chen CM. The Role of Oxidative Stress in Parkinson's Disease. *Antioxidants (Basel, Switzerland)* 2020;9(7) doi: 10.3390/antiox9070597 [published Online First: 2020/07/12]

47. Dias V, Junn E, Mouradian MM. The role of oxidative stress in Parkinson's disease. *Journal of Parkinson's disease* 2013;3(4):461-91. doi: 10.3233/jpd-130230 [published Online First: 2013/11/21]

48. Smeyne M, Smeyne RJ. Glutathione metabolism and Parkinson's disease. *Free radical biology & medicine* 2013;62:13-25. doi: 10.1016/j.freeradbiomed.2013.05.001 [published Online First: 2013/05/15]

49.. Liu YAowu, Liu Wanbin, Feng Xiaoyi. Effects of station piling on superoxide dismutase in

For peer review only - http://bmjopen.bmj.com/site/about/guidelines.xhtml

human red blood cells. *Journal of Shaanxi College of Traditional Chinese Medicine.* 1991(01):27

50. Kadkhodaie M, Sharifnezhad A, Ebadi S. Effect of eccentric-based rehabilitation on hand tremor intensity in Parkinson disease. *Neurological sciences : official journal of the Italian Neurological Society and of the Italian Society of Clinical Neurophysiology* 2020;41(3):637-43. doi: 10.1007/s10072-019-04106-9 [published Online First: 2019/11/19]

51. Zach H, Dirkx MF, Pasman JW. Cognitive Stress Reduces the Effect of Levodopa on Parkinson's Resting Tremor. *CNS neuroscience & therapeutics* 2017;23(3):209-15. doi: 10.1111/cns.12670 [published Online First: 2017/01/11]

52. Lee HJ, Lee WW, Kim SK. Tremor frequency characteristics in Parkinson's disease under resting-state and stress-state conditions. *Journal of the neurological sciences* 2016;362:272-7. doi: 10.1016/j.jns.2016.01.058 [published Online First: 2016/03/06]

53. Wang Jia-qiu. Functional magnetic resonance study on the relationship between motor and cognition in different subtypes of Parkinson's disease [PhD]. Zhejiang University, 2018.

54. Shao Y, Le W. Recent advances and perspectives of metabolomics-based investigations in Parkinson's disease. *Molecular neurodegeneration* 2019;14(1):3. doi: 10.1186/s13024-018-0304-2 [published Online First: 2019/01/13]

55. Adjustment of three-circle standing pile on mental health level of medical college students. 30th anniversary of world Medical Qigong Association; 2019; Beijing, China.

For peer review only - http://bmjopen.bmj.com/site/about/guidelines.xhtml

56. Huang Bingshuang. Research on the interference of Station post and Wuxing Music on College Students' attention [Master's degree]. Guangxi University for Nationalities, 2017.

57. The effect of exercise exercise on cognitive control (ERP Study). 2014 China Sports Physiology and Biochemistry Academic Conference; 2014; Guiyang, Guizhou Province, China.

58. Yang Si-huan, Yang Qin-fei, Shi Ji-ming. J J CHINESE JOURNAL OF INTEGRATED TRADITIONAL AND WESTERN MEDICINE. 1994(11):643-46.

59. Fang Chao. Study on intervention of regimen pile on mild and moderate hypertension [Master's degree]. *Beijing Sport University*, 2014.

60. Ma JIanguo. Experimental study on the brain mechanism of Yi Quan (Dacheng Quan) station post promoting emotion and Executive Control (NEAR Infrared Spectroscopy) [Master. *Capital Institute of Physical Education*, 2011.

61. Li Chun-ri, Ren Da-peng, Bai Zeng-hua. Change of integral emG of taijiquan pole post in initial stage. *Journal of Practical Traditional Chinese Medicine*. 2013; 27 (08) : 13-14.

62. Liu Xiaoli. Preliminary study on physiological mechanism of Tai Chi station pile. *J FUJIAN SPORTS SCIENCE AND TECHNOLOGY*. 1996(4):20-21,37.

63. Wu Dong, XIONG Kaiyu, Song Yuan. Study on electromyographic changes of xingyiquan Three-body potential station pile. *Journal of Beijing Sport University*. 2012; 35 (4) : 65-69 + 73

64. Zhou Xue-zhu, ZHANG Zhi-xing, CUI Hong-jian. Study on emG changes of core muscle

For peer review only - http://bmjopen.bmj.com/site/about/guidelines.xhtml

group surface by post work. *Journal of Liaoning University of Traditional Chinese Medicine.* 2018; 20 (11) : 66-69.

65. Yang Si-huan, Yang Qin-fei, SHI Ji-ming, CAO Yi. Observation on the change of eeg frequency spectrum of exercisers. *Chinese Journal of Integrated Traditional and Western Medicine.* 1994(11):643-46.

For peer review only

For peer review only - http://bmjopen.bmj.com/site/about/guidelines.xhtml

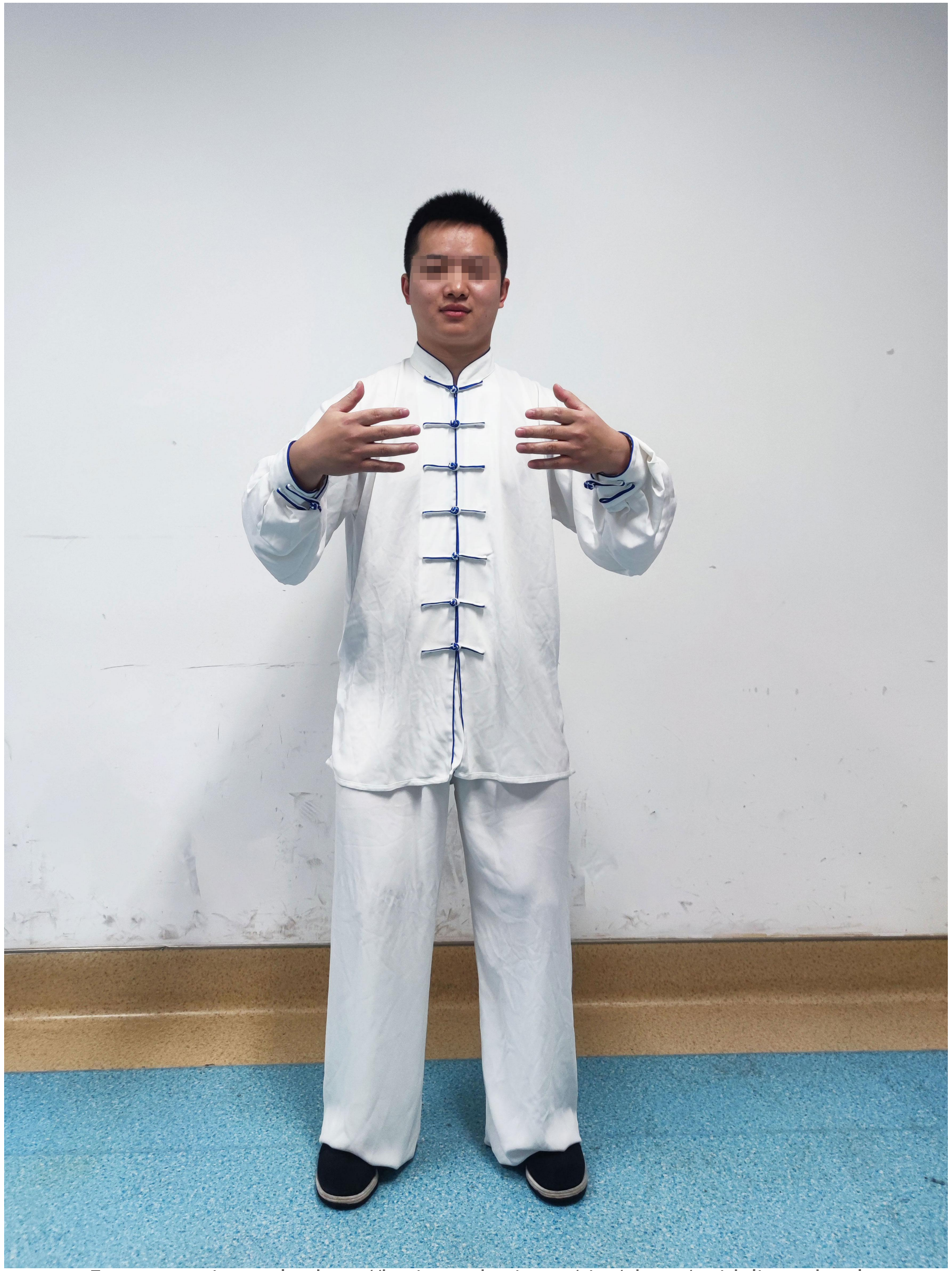

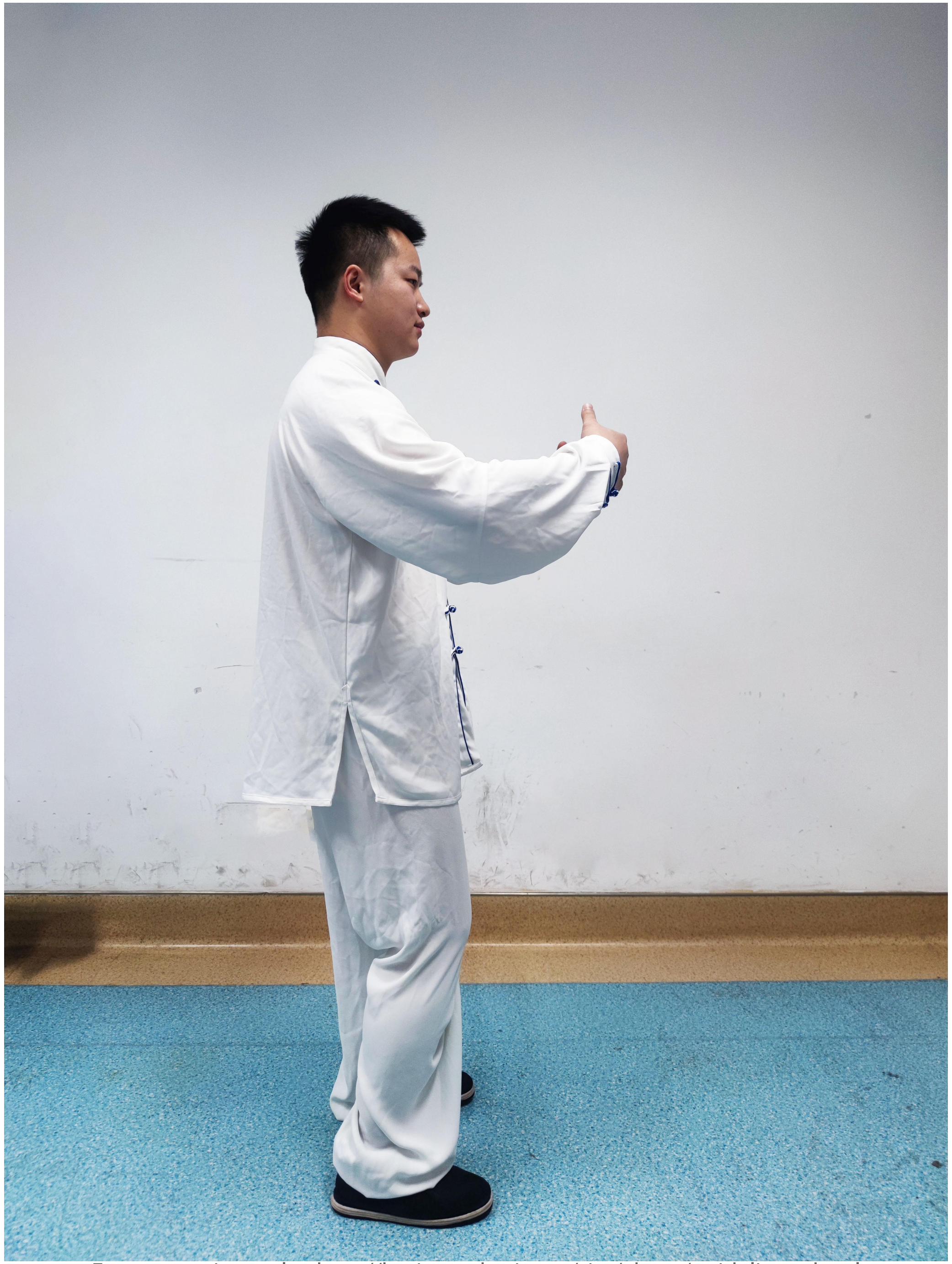

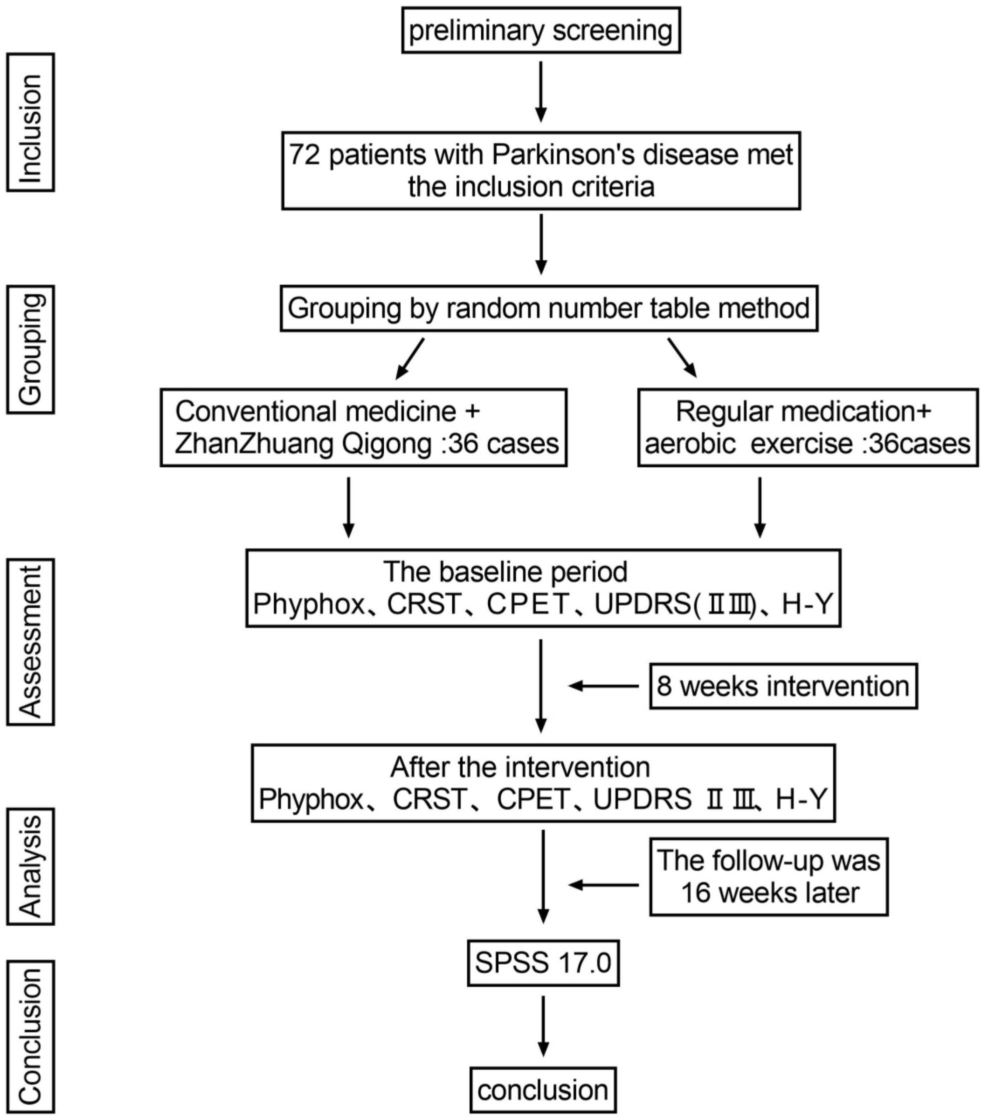

figure 3

1417x1605mm (72 x 72 DPI)

I, the Submitting Author has the right to grant and does grant on behalf of all authors of the Work, an exclusive licence and for contributions from authors where BMJ has agreed a CC-BY licence shall apply, and/or ii) in accordance with the terms applicable for US Federal Government officers or employees acting as part of their official duties; on a worldwide, perpetual, irrevocable, royalty-free basis to BMJ Publishing Group Ltd ("BMJ") its licensees and where the relevant Journal is co-owned by BMJ to the co-owners of the Journal, to publish the Work in BMJ Open and any other BMJ products and to exploit all rights, as set out in our licence.

For peer review only

Reporting checklist for protocol of a clinical trial.

Based on the SPIRIT guidelines.

Instructions to authors

Complete this checklist by entering the page numbers from your manuscript where readers will find each of the items listed below.

Your article may not currently address all the items on the checklist. Please modify your text to include the missing information. If you are certain that an item does not apply, please write "n/a" and provide a short explanation.

Upload your completed checklist as an extra file when you submit to a journal.

In your methods section, say that you used the SPIRITreporting guidelines, and cite them as:

Chan A-W, Tetzlaff JM, Gøtzsche PC, Altman DG, Mann H, Berlin J, Dickersin K, Hróbjartsson A, Schulz KF, Parulekar WR, Krleža-Jerić K, Laupacis A, Moher D. SPIRIT 2013 Explanation and Elaboration: Guidance for protocols of clinical trials. BMJ. 2013;346:e7586

		Reporting Item	Page Number
**Administrative information**			
Title	#1	Descriptive title identifying the study design, population, interventions, and, if applicable, trial acronym	
Trial registration	#2a	Trial identifier and registry name. If not yet registered, name of intended registry	
Trial registration: data set	#2b	All items from the World Health Organization Trial Registration Data Set	
Protocol version	#3	Date and version identifier	
Funding	#4	Sources and types of financial, material, and other support	
Roles and responsibilities: contributorship	#5a	Names, affiliations, and roles of protocol contributors	

For peer review only

Roles and responsibilities: sponsor contact information	#5b	Name and contact information for the trial sponsor
Roles and responsibilities: sponsor and funder	#5c	Role of study sponsor and funders, if any, in study design; collection, management, analysis, and interpretation of data; writing of the report; and the decision to submit the report for publication, including whether they will have ultimate authority over any of these activities
Roles and responsibilities: committees	#5d	Composition, roles, and responsibilities of the coordinating centre, steering committee, endpoint adjudication committee, data management team, and other individuals or groups overseeing the trial, if applicable (see Item 21a for data monitoring committee)
**Introduction**		
Background and rationale	#6a	Description of research question and justification for undertaking the trial, including summary of relevant studies (published and unpublished) examining benefits and harms for each intervention
Background and rationale: choice of comparators	#6b	Explanation for choice of comparators
Objectives	#7	Specific objectives or hypotheses
Trial design	#8	Description of trial design including type of trial (eg, parallel group, crossover, factorial, single group), allocation ratio, and framework (eg, superiority, equivalence, non-inferiority, exploratory)
**Methods: Participants, interventions, and outcomes**		
Study setting	#9	Description of study settings (eg, community clinic, academic hospital) and list of countries where data will be collected. Reference to where list of study sites can be obtained
Eligibility criteria	#10	Inclusion and exclusion criteria for participants. If applicable, eligibility criteria for study centres and individuals who will

For peer review only

		perform the interventions (eg, surgeons, psychotherapists)
Interventions: description	#11a	Interventions for each group with sufficient detail to allow replication, including how and when they will be administered
Interventions: modifications	#11b	Criteria for discontinuing or modifying allocated interventions for a given trial participant (eg, drug dose change in response to harms, participant request, or improving / worsening disease)
Interventions: adherance	#11c	Strategies to improve adherence to intervention protocols, and any procedures for monitoring adherence (eg, drug tablet return; laboratory tests)
Interventions: concomitant care	#11d	Relevant concomitant care and interventions that are permitted or prohibited during the trial
Outcomes	#12	Primary, secondary, and other outcomes, including the specific measurement variable (eg, systolic blood pressure), analysis metric (eg, change from baseline, final value, time to event), method of aggregation (eg, median, proportion), and time point for each outcome. Explanation of the clinical relevance of chosen efficacy and harm outcomes is strongly recommended
Participant timeline	#13	Time schedule of enrolment, interventions (including any run-ins and washouts), assessments, and visits for participants. A schematic diagram is highly recommended (see Figure)
Sample size	#14	Estimated number of participants needed to achieve study objectives and how it was determined, including clinical and statistical assumptions supporting any sample size calculations
Recruitment	#15	Strategies for achieving adequate participant enrolment to reach target sample size
**Methods: Assignment of interventions (for controlled trials)**		
Allocation: sequence generation	#16a	Method of generating the allocation sequence (eg, computer-generated random numbers), and list of any factors for stratification. To reduce predictability of a random sequence, details of any planned restriction (eg, blocking) should be provided in a separate document that is unavailable to those who enrol participants or assign interventions

For peer review only

Allocation concealment mechanism	#16b	Mechanism of implementing the allocation sequence (eg, central telephone; sequentially numbered, opaque, sealed envelopes), describing any steps to conceal the sequence until interventions are assigned
Allocation: implementation	#16c	Who will generate the allocation sequence, who will enrol participants, and who will assign participants to interventions
Blinding (masking)	#17a	Who will be blinded after assignment to interventions (eg, trial participants, care providers, outcome assessors, data analysts), and how
Blinding (masking): emergency unblinding	#17b	If blinded, circumstances under which unblinding is permissible, and procedure for revealing a participant's allocated intervention during the trial
**Methods: Data collection, management, and analysis**		
Data collection plan	#18a	Plans for assessment and collection of outcome, baseline, and other trial data, including any related processes to promote data quality (eg, duplicate measurements, training of assessors) and a description of study instruments (eg, questionnaires, laboratory tests) along with their reliability and validity, if known. Reference to where data collection forms can be found, if not in the protocol
Data collection plan: retention	#18b	Plans to promote participant retention and complete follow-up, including list of any outcome data to be collected for participants who discontinue or deviate from intervention protocols
Data management	#19	Plans for data entry, coding, security, and storage, including any related processes to promote data quality (eg, double data entry; range checks for data values). Reference to where details of data management procedures can be found, if not in the protocol
Statistics: outcomes	#20a	Statistical methods for analysing primary and secondary outcomes. Reference to where other details of the statistical analysis plan can be found, if not in the protocol
Statistics: additional analyses	#20b	Methods for any additional analyses (eg, subgroup and adjusted analyses)

For peer review only

Statistics: analysis population and missing data	#20c	Definition of analysis population relating to protocol non-adherence (eg, as randomised analysis), and any statistical methods to handle missing data (eg, multiple imputation)
**Methods: Monitoring**		
Data monitoring: formal committee	#21a	Composition of data monitoring committee (DMC); summary of its role and reporting structure; statement of whether it is independent from the sponsor and competing interests; and reference to where further details about its charter can be found, if not in the protocol. Alternatively, an explanation of why a DMC is not needed
Data monitoring: interim analysis	#21b	Description of any interim analyses and stopping guidelines, including who will have access to these interim results and make the final decision to terminate the trial
Harms	#22	Plans for collecting, assessing, reporting, and managing solicited and spontaneously reported adverse events and other unintended effects of trial interventions or trial conduct
Auditing	#23	Frequency and procedures for auditing trial conduct, if any, and whether the process will be independent from investigators and the sponsor
**Ethics and dissemination**		
Research ethics approval	#24	Plans for seeking research ethics committee / institutional review board (REC / IRB) approval
Protocol amendments	#25	Plans for communicating important protocol modifications (eg, changes to eligibility criteria, outcomes, analyses) to relevant parties (eg, investigators, REC / IRBs, trial participants, trial registries, journals, regulators)
Consent or assent	#26a	Who will obtain informed consent or assent from potential trial participants or authorised surrogates, and how (see Item 32)
Consent or assent: ancillary studies	#26b	Additional consent provisions for collection and use of participant data and biological specimens in ancillary studies, if applicable
Confidentiality	#27	How personal information about potential and enrolled participants will be collected, shared, and maintained in order to protect confidentiality before, during, and after the trial

For peer review only

Declaration of interests	#28	Financial and other competing interests for principal investigators for the overall trial and each study site
Data access	#29	Statement of who will have access to the final trial dataset, and disclosure of contractual agreements that limit such access for investigators
Ancillary and post trial care	#30	Provisions, if any, for ancillary and post-trial care, and for compensation to those who suffer harm from trial participation
Dissemination policy: trial results	#31a	Plans for investigators and sponsor to communicate trial results to participants, healthcare professionals, the public, and other relevant groups (eg, via publication, reporting in results databases, or other data sharing arrangements), including any publication restrictions
Dissemination policy: authorship	#31b	Authorship eligibility guidelines and any intended use of professional writers
Dissemination policy: reproducible research	#31c	Plans, if any, for granting public access to the full protocol, participant-level dataset, and statistical code

Appendices

Informed consent materials	#32	Model consent form and other related documentation given to participants and authorised surrogates
Biological specimens	#33	Plans for collection, laboratory evaluation, and storage of biological specimens for genetic or molecular analysis in the current trial and for future use in ancillary studies, if applicable

None The SPIRIT Explanation and Elaboration paper is distributed under the terms of the Creative Commons Attribution License CC-BY-NC. This checklist can be completed online using https://www.goodreports.org/, a tool made by the EQUATOR Network in collaboration with Penelope.ai

For peer review only

BMJ Open

Effect of ZhanZhuang Qigong on upper limb static tremor and aerobic exercise capacity in patients with mild to moderate Parkinson's disease: Study Protocol for a Randomized controlled Trial

Journal:	*BMJ Open*
Manuscript ID	bmjopen-2021-059625.R1
Article Type:	Protocol
Date Submitted by the Author:	08-Mar-2022
Complete List of Authors:	zhang, linlin; Shandong University of Traditional Chinese Medicine,<br>Liu, Xihua; Shandong University of Traditional Chinese Medicine Affiliated Hospital<br>Xi, Xiaoming; Shandong University of Traditional Chinese Medicine<br>Chen, Yuxiao; Shandong University of Traditional Chinese Medicine<br>Wang, Qing; Shandong University of Traditional Chinese Medicine<br>Qu, Xinjie; Shandong University of Traditional Chinese Medicine<br>Cao, Haihao; Shandong University of Traditional Chinese Medicine<br>Wang, Limin; Shandong University of Traditional Chinese Medicine<br>Sun, Wenyu; Shandong University of Traditional Chinese Medicine Affiliated Hospital<br>Chen, Guoming; Shandong University of Traditional Chinese Medicine Affiliated Hospital<br>Liu, Huifen; Shandong University of Traditional Chinese Medicine<br>Jiang, Xiaoyu; Shandong University of Traditional Chinese Medicine<br>Su, Hang; Shandong University of Traditional Chinese Medicine<br>Jiang, Jiahui; Shandong University of Traditional Chinese Medicine<br>Bi, Hongyan; Shandong University of Traditional Chinese Medicine Affiliated Hospital
**Primary Subject Heading**:	Geriatric medicine
Secondary Subject Heading:	Rehabilitation medicine, Neurology
Keywords:	Parkinson-s disease < NEUROLOGY, GERIATRIC MEDICINE, Rehabilitation medicine < INTERNAL MEDICINE, Clinical trials < THERAPEUTICS

SCHOLARONE™
Manuscripts

Effect of ZhanZhuang Qigong on upper limb static tremor and aerobic exercise capacity in patients with mild to moderate Parkinson's disease: Study Protocol for a Randomized controlled Trial

Linlin Zhang, MD[1]; Xihua Liu, Ph. D[2]; Xiaoming Xi, MD[1]; Yuxiao Chen, MD[1]; Qing Wang, MD[1]; Xinjie Qu, MD[1]; Haihao Cao[1], MD[1]; Limin Wang[1], MD[1]; Wenyu Sun, MD[2]; Guoming Chen, MD[2]; Huifen Liu, MD[1]; Xiaoyu Jiang, MD[1]; Hang Su, MD[1]; Jiahui Jiang, MD[1]; Hongyan Bi, MD[1].

1: Shandong University of Traditional Chinese Medicine, Jinan, China

2: Shandong University of Traditional Chinese Medicine Affiliated Hospital, Jinan, China

Correspondence

Hongyan Bi, Shandong University of Traditional Chinese Medicine Affiliated Hospital, Jinan, China

Tel: 18800460276

Email: Hy__bi@163.com

Abstract

Introduction: Currently, the first choice for the clinical treatment of static tremor in Parkinson's disease (PD) is drug therapy, however side effects are common. In recent years, the effects of physical therapy on PD has become a serious research focus. Studies have indicated that aerobic and resistance exercises alleviate PD movement disorders and improve aerobic capacity, but the effects of Qigong on PD static tremor and aerobic capacity remain unknown.

Methods and analysis

Objective: To observe the effects of Zhanzhuang Qigong on upper limb static tremor and aerobic capacity in patients with PD, we established a rigorous randomized, parallel-controlled, assignment hidden, evaluator-blinded protocol. **Methods**: Seventy-two patients with PD, at the Affiliated Hospital of Shandong University of Traditional Chinese Medicine, were recruited and randomly divided into a control (n = 36) and experimental group (n = 36). The intervention group received conventional medicine plus ZhanZhuang Qigong exercises 5 times a week at 30 min each time, over an 8 week period. The long-term effects of ZhanZhuang Qigong on PD was investigated after the intervention. Phyphox APP, CRST, CPET, UPDRS (II, III) were used to evaluate tremor, aerobic capacity， and motor function ingroups. **Discussion**: we are investigating the effects of ZhanZhuang Qigong on upper limb static tremor and aerobic capacity in PD patients. If positive are identified, they will add a new research direction and evidence for the clinical exploration of exercise therapy for PD.

Ethics and dissemination

This study was approved by the Ethics Committee of Shandong University of Traditional Chinese Medicine (Approval Number: 2021-025-KY). The Committee will be informed of any changes to the trial protocol, such as intervention intensity, outcome indicators and data collection. Study results will be presented as a paper at an international conference or in a journal.

For peer review only - http://bmjopen.bmj.com/site/about/guidelines.xhtml

Test registration Number

The study protocol was registered in the Chinese Clinical Trial Registry (Registration number: ChiCTR2100053529)

Version:

20211124

Key words: ZhanZhuang Qigong, Parkinson's disease, tremor, aerobic exercise capacity

Strengths and limitations of this study

1. Currently, the clinical effects of physical therapy toward Parkinson's tremor are not ideal, with no clinical studies conducted on the effects of Qigong on Parkinson's tremor.
2. The mobile phone tremor test and cardiopulmonary exercise test used in our outcome assessments were clinically verified as accurate and reliable.
3. Our rigorous hidden allocation, randomized parallel controlled trial design is more reliable and accurate than pre-and post-trial controls previously used in PD clinical trials.
4. Staff turnover during the study intervention period may lead to bias in the final results.
5. we are unable to investigate other forms of Parkinson's tremor other than static tremor.

Introduction

Parkinson's disease (PD) is a degenerative disease of the central nervous system common in middle-aged and elderly individual[1]. The PD prevalence in individuals aged 65-69 is approximately 0.5%-1%, and in individuals over 80, 1-3%. With increased population aging, it is anticipated that PD prevalence and incidence will increase by more than 30% by 2030, and place tremendous pressure on families and society[2].

As one of the main motor symptoms of PD, static tremor is characterized by rhythmic and clay ball-like tremor and is accompanied by a large amplitude and frequency of 4-6Hz, which occurs more when the weight of affected limbs or other body structures is supported. The tremor is aggravated when general exercises (such as walking) or pressures increases, but is alleviated or disappears when the body moves at will. The tremor gradually spreads from the distal end of one limb to the other[3 4]. Parkinson's tremor is one of the most troubling symptoms in patients with PD, which not only reduces daily living activities, but also imbues a sense of inferiority, depression, and reduces participation in society[5]. Currently, the most effective clinical treatment for PD remains drug therapy[2 6 7]. Levodopa is considered the best treatment compound for supplementing exogenous dopamine and controlling PD, but its long-term use gradually reduces its efficacy and leads to central and peripheral adverse reactions[8-10]. Moreover, levodopa elicits poor responses to PD static tremor and has no effect on non-motor symptoms[11]. Invasive surgical treatments, such as focused thalamotomy and deep brain stimulation provide significant tremor relief in the short term, but do not prevent neurodegenerative progression in underlying PD[8 12-14]. Additionally, surgery is risky and may be accompanied by complications[12].

In recent years, PD symptom improvement by exercise therapy has gained considerable research traction. Several studies reported that aerobic and resistance

For peer review only - http://bmjopen.bmj.com/site/about/guidelines.xhtml

exercises alleviated PD motor symptoms, including tremor[15-19]. Among traditional Chinese Qigong therapies, Tai Chi, Wuqinxi and Baduanjin are believed to enhance balance in PD patients and correct abnormal gait. However, few studies have focused on PD tremor and aerobic capacity[20].

ZhanZhuang Qigong is a basic technique in Tai Chi, and is a static, moderate, and low intensity aerobic exercise combining body and mind[21 22]. Several clinical studies have reported the benefits of Tai Chi for PD motor and non-motor functions[23], Tai Chi is a low cost exercise, it reduces falls, improves posture control, walking ability, improves depression and insomnia symptoms, and improve quality of life. As a basic Tai Chi skill, the ZhangZhuang has many Tai Chi characteristics[24]. For example, ZhanZhuang movements combine body and mind, they control limb and trunk stability and change the center of gravity with the mind. Additionally, like Tai Chi, ZhanZhuang is also a closed circular motion technique which maintains action continuity and accuracy via adjustment, feedback, re-adjustment, and re-feedback, which improve the body's recognition of stimuli and improves involuntary tremor[25 26].

Methods and analysis

The study is a two-arm, randomized, parallel-controlled trial with assignment-hiding and evaluator blindness. The main objective is to investigate the effects of ZhanZhuang on upper limb static tremor and aerobic capacity in patients with mild to moderate PD, so as to provide a safe, effective, low-cost, and easy-to-master aerobic exercise therapy for PD patients.

Patient and public involvement

The public is not involved in the study design. However, we will adjust intervention intensity and other details based on how participants respond to the intervention.

Study design and setting

The study will be conducted in the Affiliated Hospital of Shandong University of Traditional Chinese Medicine. We will include 72 patients with PD with obvious upper limb static tremor. Recruitment will start from 1 June 2022, until when 72 participants are enrolled. Using the random number table method, 72 patients will be randomly divided into an intervention and control group in a 1:1 ratio. Basic patient information, including gender, age, disease course, education level, residence status and drug-use will be collected. Tremor and motor function tests will be performed at least 8 hours after patients stop taking medication.

The control group will be treated with conventional medicine and routine aerobics. According to the Chinese Guidelines for the Treatment of PD (4th edition), walking will be selected as the aerobic exercise mode in this group[27], five times a week at 30 min each time for 8 weeks. A combined cardiopulmonary exercise test (CPET) (treadmill test) will be used to measure peak oxygen uptake (peakVO_2) in PD patients at baseline and after intervention, and also anaerobic threshold (AT) and maximum heart rate (HRmax). During training, patients exercise intensity will be controlled at moderate exercise intensity (HRmax (60%-80%) measured by CPET or 11-13 self-induced Fatigue Scale (RPE)).

For peer review only - http://bmjopen.bmj.com/site/about/guidelines.xhtml

Changes in peakVO_2 and AT at baseline and after intervention will be evaluated for improvements in aerobic exercise capacity. Walking will be performed about 1 after patients take their medication, and the tremor symptoms were resolved or disappeared.

The intervention group will take conventional medicine combined with ZhanZhuang Qigong. An experienced Qigong master will guide ZhanZhuang exercise. Week 1 will comprise the learning phase, while weeks 2-8 will be the practice phases, to be performed 5 times a week at 30 min each time. Intensity HRmax (60%-80%) will be measured using a cardiopulmonary combined exercise test or RPE test grade 11-13. Patients will be issued a training instruction manual and a personal exercise calendar to record the number of weekly exercises and time of each exercise. ZhanZhuang training will be about 1 h after patients take their medication, and tremor symptoms were relieved or disappeared. One auxiliary staff member will oversee safety measures.

The main points of Qigong movement of ZhanZhuang:

Individuals should stand naturally, with feet about 30 cm apart, and parallel to the front. Arms around, with naturally open hands and two fingertips about 15 cm apart, Flex the knee hip about 5-10° and tilt the hip forward (Figures 1, 2).

Participants and Recruitment

Using the Affiliated Hospital of Shandong University of Traditional Chinese Medicine as the base, we will visit and recruit patients from the rehabilitation and neurology departments, and post recruitment advertisements on Wechat.

We will assess upper limb static tremor according to the Unified Parkinson's Disease Rating Scale (UPDRS) Part III and the Hoehn-Yahr classification (stages 1-3) (see Figure 3).

Inclusion criteria

(1) Primary PD agrees with recommended Chinese Clinical Diagnostic Criteria for PD (2016);
(2) 50-70 years old;
(3) Improved Hoehn-Yahr stage 1-3;
(4) Stable condition, taking Madopa, and standing for the "prescribed period";
(5) No serious cognitive dysfunction, has the ability to learn ZhanZhuang movements; (Mini-Mental State Examination (MMSE) score, illiteracy > 17, primary school > 20 and middle school > 22);
(6) Agrees to participate, and sign an informed consent sheet.

Exclusion criteria:

(1) Secondary Parkinson's syndrome and Parkinson's superposition syndrome caused by cerebrovascular disease, drugs, and other causes;
(2) Patients with serious heart, lung, liver, kidney dysfunction or cancer;
(3) Those individuals who cannot stand due to serious knee diseases, e.g., severe knee osteoarthritis and knee trauma;

For peer review only - http://bmjopen.bmj.com/site/about/guidelines.xhtml

(4) Patients with dementia or serious mental illness;
(5) Patients unable to stand or walk independently due to fracture, surgery, or other reasons;
(6) No tremors;
(7) The "opening period" tremor is obvious after taking medication.

Termination/shedding criteria
(1) Patients not cooperating with prescribed exercises or exercising poor compliance;
(2) Patients whose condition deteriorates or complications arise during the study should not continue.

Sample size

This study is designed as a randomized controlled trial. The main outcome is the mean relief of upper limb static tremor after treatment. A review of previous studies showed that aerobic exercise could reduce upper limb static tremor amplitude by approximately 60%. It is estimated the average tremor amplitude of the control group is approximately 76 ± 136 mm, and the intervention group is expected to be reduced by 50 mm after training. The test level α is set at 0.05 (bilateral), the degree of certainty 1-β is set at 0.80, and the control: intervention group ratio = 1:1. A sample size of 63 was calculated using *http://powerandsamplesize.com*. However, considering an abscission rate of about 15%, 72 patients with PD will be included.

Randomization and Allocation Concealment and Blinding

This study is single-blinded. Because of the Qigong intervention, training personnel and subjects are not blinded, however testers, data collectors, data scientists, and analysts are blinded. Implementation of training, evaluation and statistical analysis of the separation of the way, that is, the training personnel do not contact the test personnel. Before testing, the intervention and control groups were renamed as groups A and B, respectively. The test tester is only responsible for the test by number, and does not tell the group of the test subject. Data collection and processing personnel are separated; data collection personnel obtain group A or group B date based on the number and prove this information to the data statistician for statistical analysis. To ensure study objectivity, quality control personnel will identify groups after statistics are completed.

Assessment result

Primary outcome

(1) Tremor test

① Mobile phone movement data collection

Phyphox APP[28] is based on the Android system (version: 1. 1. 9). It collects the static tremor date of the upper limb of patients with an obvious tremor, at a frequency of 100Hz over 30 s.

Data collection on the static tremor: The patient relaxes, sits in a chair, places his/her hands on the armrest, and holds the mobile phone with the screen up on the shaking side. At the same time, patients perform numerical subtraction calculations to distract their

For peer review only - http://bmjopen.bmj.com/site/about/guidelines.xhtml

attention. The time from the beginning of the tremor is recorded over 30 s.

② The Clinical Tremor Rating Scale (CRST) A+B, CRST-A is used to evaluate the tremor at rest, postural, and intentional; CRST-B assesses handwriting (dominant hand only), wide and narrow spiral drawing, straight lines, and casting. For the dominant hand, the maximum sub-score of hand tremor is 32 for CRST (A+B) and 28 for the non-dominant hand. The higher the score, the more severe the tremor.

(2) Combined Cardiopulmonary Exercise Test (CPET)

The cardiovit CS-200 cardiopulmonary combined exercise test system (SCHILLERA, Switzerland) is used for CPET. The treadmill test is performed, and the increasing rate set at 10W/min. Based on standards from Harbor-UCLA Center Cardiopulmonary Exercise Laboratory, USA, the resting state lung function examination, including fast lung capacity, slow lung capacity, maximum minute ventilation capacity, and diffusion function, should be completed first. Symptom limiting maximum CPET is then completed using continuous ramping regimen[29-31]. CPET is divided into four stages, including 3 min of rest, 3 min warm-up (no treadmill power), then exercise at an increasing power rate of 10W/min until symptom limiting maximum exercise, followed by more than 5min of recovery[32]. Continuous 12-lead ECG, non-invasive cuff blood pressure, and oxygen saturation measurements are required during the CPET process. PeakVO_2, AT, and HRmax are measured and recorded.

(3) Assessing motor symptoms and daily living abilities

(1) Unified UPDRS IIandIII: The scale is divided into four parts. The first part evaluates the patient's mental behaviors and emotions. Part II assesses the patient's ability to live a daily life. Part III assesses the patient's motor functions; The total score in part Ⅳ reflects patient complications. We selected PartsIIand part III to evaluate the daily living activities and motor functions of patients, respectively.

(2) Revised Hoehn-Yahr Grading Scale: The scale is divided into eight stages, from 0-5 (0, 1, 1.5, 2, 2.5, 3, 4 and 5), to evaluate disease severity.

Adverse events (AEs)

AEs occurring during the study are recorded using a Case Report Form. For this trial, an AE will be defined as adverse and unexpected signs, symptoms or diseases associated with the intervention, such as falls, joint injuries, hypertension, etc. Participants will be asked about their medical history and any AEs they have experienced before training begins, and AEs will be recorded after each intervention. The principal investigator will be notified within 24 h if a serious adverse event (SAE) occurs. If subjects withdraw from the trial due to any AE, the study team will follow-up accordingly or until the AE is resolved, This information will be recorded in the original file. The principal investigator manages all safety reports. Any AEs relating to the intervention will be reported to the Ethics Review Committee of the Affiliated Hospital of Shandong University of Chinese Medicine.

For peer review only - http://bmjopen.bmj.com/site/about/guidelines.xhtml

For peer review only

Statistical analysis

We will evaluate the outcome and collect data at week 9 of the intervention and follow up at week 17 of the intervention. (Table 1) . Basic patient information, mobile phone exercise test data, CRST scores, and UPDRS（II，III）scores will be exported to our computer system CSV format.

SPSS17.0 statistical software will be used for data processing and analysis: 1). Measurement data will be processed using the mean standard deviation ($x^2 \pm s$); 2). T tests will be used assess: normal distribution and homogeneity of the overall variance in both samples; 3). T tests will be used to assess: normal distributions, but uneven overall variance; 4). The Wilcoxon rank sum test will assess if date does not conform to a normal distribution; $P < 0.05$ indicates statistical significance.

Mobile exercise test data

The original form is the acceleration a (m/s) of three axes (X, Y, and Z axes) vector direction respectively), rotation radian w (rad/s). The MATLAB (version R 2018a, MathWorks, Inc, USA) computer program will be used to perform Fourier transformation of the data, and acceleration and rotation radian measurements by smart phone will be converted to corresponding frequency, peak amplitude, and speed date. Final phone test data will be presented as the amplitude and frequency of static tremors. Then, normal distribution tests will be conducted. Since the sample size is small (N < 5000), the Shapiro-Wilk normal test will be used. $P > 0.05$ indicates a normal distribution, while $P < 0.05$ indicates a non-normal distribution.

According to normal distribution test date, if mobile phone exercise test data is normally distributed and the variance uniform, mobile phone exercise test data in PD patients in different groups will be tested using with two sample independent T tests. $P < 0.05$ will indicated if the exercise is statistically significant between groups. If the data are not normally distributed, non-parametric tests (median and Mann-Whitney U tests) will be performed for comparison. $P < 0.05$ in median tests will indicate if differences in central tendency scores between groups are statistically significant.

CPET test data

After subjects complete the CPET test, raw date will be saved. According to standardized principles of CPET data analysis, and the interpretation of FDA clinical trials in the Harbor-UCLA Medical Center[33 34], original breath-by-breath data from subjects will be segmsed every second from the CPET system software. Then, average the data for 10s and export the average data[31 35]. The warm-up value is the average of the last 30s of the warm-up period, the anaerobic threshold value is the data value of 10s when the anaerobic threshold, and the peak value is the average of 30s during the extreme exercise. Anaerobic threshold analysis is performed using a v-slope method, i.e., the mean value of 10s of oxygen uptake when the CO_2 emission rate is significantly accelerated relative to oxygen uptake rate during movement[36].

Discussion

PD not only reduces patients quality of life, but also generates a heavy economic

For peer review only - http://bmjopen.bmj.com/site/about/guidelines.xhtml

burden for society and families. Upper limb static tremor is one of the main PD motor symptoms. Dyskinesia may can also lead to decreased activity and aerobic capacity in PD patients. Currently, non-drug therapy for PD mainly includes aerobic exercise and resistance training. Previous studies reported those methods were beneficial for tremor, bradykinesia, balance, and quality of life in PD patients[37]. However, the effect of Qigong on PD static tremor and aerobic ability remains unknown.

Free radicals (OH)[38] are a strong oxides that induce lipid peroxidation in unsaturated fatty acids and produce lipid peroxidation (LPO), which damages proteins and DNA and lead to cell degeneration and death. Oxidative stress (OS)[39] represents an imbalanced state between oxidation and antioxidant effects in the body, which are important factors contributing to aging and disease. Due to increased activity of B-monoamine oxidase (MAO-B) in PD patients, excessive free radicals oxidize and destroy cell membranes[40]. At the same time, dopamine oxidation products in substantia nigra cells polymerize to form neuromelanin, which combines with iron to produce Fenton reactions and free radicals. Under normal circumstances, antioxidant molecules in brain cells are plentiful and include, glutathione (GSH), glutathione peroxidase (GSH-PX), and superoxide dismutase (SOD), thus free radicals generated by dopamine oxidation will not lead to oxidative stress[41 42]. In PD, the substantia nigra is vulnerable to oxidative stress due to decreased ferritin and glutathione leves, and increased lipid peroxide, and iron ion concentrations[41-43]. Previous studies[11 44] reported that ZhanZhuang improved SOD the activity levels in the body, prevented harm from superoxide free radicals, and exerted positive roles fighting aging, inflammation, and immune disease, Therefore, the ZhangZhuang may protect the substantia nigra in PD patients by increasing SOD activity levels, reducing substantia nigra lesions, slowing down PD progression, and improving overall symptoms in PD patients.

Currently, the generally accepted PD tremor mechanism in clinical practice is the induced-switch-regulation model proposed by Duval[4].

Induced: Pathological activity in the basal nucleus striatum induces rhythmic cluster discharge activity in thalamic neurons via he excessive input of inhibitory signals to thalamic cells, and changes the discharge mode.

Switch: Oscillations in internal and external networks of the thalamus combine and cause oscillations to continuously integrate and strengthen until they reach tremor production threshold values.

Regulation: After PD tremor-related oscillations are introduced to the cerebellum, it processes oscillation as random movements and adjusts tremors to ensure stability in oscillation activities, such as frequency and amplitude. Tremors transmitted to the cerebral cortex are then transmitted to the periphery via the cortical-spinal cord pathway in the form of oscillations, and are finally manifested body tremors. Yang *et al.*[45], based on data from basal ganglia injury mice and rat models, reported that exercise training reduced over-activation of basal ganglia cortical circuits, thereby weakening tremor signal transmission, and alleviating tremors. Recent studies also reported these animal models were applicable to humans[46]. Mona *et al.*[15] observed that upper limb centrifugal exercises effectively reduced upper limb tremor amplitude in PD disease patients. ZhanZhuang as an aerobic exercise may also reduce tremors at the motor level.

PD tremor is influenced by attention and stress levels[47]. Previous studies reported that

For peer review only - http://bmjopen.bmj.com/site/about/guidelines.xhtml

tremors were intentionally attenuated when attention was focused on them, and that PD tremors were is amplified by stress[48]. Jiaqiu Wang[49] using nuclear magnetic resonance (NMR) to analyze tremor subtypes in patients with PD, observed that the brains of tremor subtypes in patients with a wide range of functional connection between the frontal lobe and cerebellum, and UPDRS score III movement and the cognitive function score also existed significant correlation, that movement function and cognitive function in patients with PD has a high degree of correlation. DA substitution therapy also alleviated some motor characteristics in PD, but the observed beneficial effects on cognitive function were small[50]. Several studies[21 51 52] reported that ZhanZhuang enhanced the attention and cognitive ability of adolescents and the elderly. Yiyue Chen[53] observed that ZhanZhuang exercises increased P3 wave amplitude in frontal, central, and top regions, with a shorter incubation period when compared with baseline. P3 wave amplitude reflected the allocation of neural electrical resources, and latency which was related to the speed of stimulus classification. These results suggest that station post affected neural electrical processing related to cognitive control , by changing neural electrical resource allocation and stimulus classification speeds, thereby affecting cognitive function. Therefore, the station post may indirectly improve tremor performance by enhancing cognitive ability in PD patients.

Executive control is a general control mechanism where individuals coordinate different cognitive processes and resources to ensure their cognitive system implements specific goals in a flexible and optimized way when completing complex cognitive tasks. Executive control also coordinates brain commands and muscle movements. Sihuan Yang[54] conducted a 1 year ZhanZhuang exercise in healthy college students, and observed that EEG activity coefficients in students who participated in ZhanZhuang exercises were generally decreased, and that EEG's showed a high synchronization trend. Whole brain ordering was improved, and brain activities were more coordinated and synchronized. ChaoFang[55] reported that ZhanZhuang can exercised the muscles of the whole body statically, provided benign stimulation to the brain, and adjusted the disorder of excitement and inhibition of the cerebral cortex. Jianguo Ma[56] also confirmed that ZhanZhuang improved subject response speeds and accuracy, and also improved the executive control levels in the brain. ZhanZhuang combines body and mind, it focuses on controlling the body with the mind, and mobilizing muscle groups in the trunk and limbs to participate in this process[21]. This body and mind combination stimulates the nervous system and increase its sensitivity, ensuring more efficient and precise muscle control. Additionally, ZhanZhuangs places the body in a stress state of postural control for long period, with previous clinical studies showing that stress significantly affects neural plasticity[56]. Thus, higher levels of executive control and finer, more efficient postural control may benefit PD tremor relief.

YU Guo[21] observed that B cell receptor and NF-κB signaling pathways were significantly enriched in college students after ZhanZhuang, suggesting the technique may enhance *in vivo* immunity and improve inflammatory microenvironments by activating B cell receptor signaling and improving T lymphocyte levels, or regulating NF-κB signaling.

For peer review only - http://bmjopen.bmj.com/site/about/guidelines.xhtml

Regulating valine, leucine, and isoleucine metabolism, prevents excessive glutamate accumulation, increases astrocyte and 5-hydroxytryptamine levels, and prevents neuronal necrosis and apoptosis. The specific regulation of the lysosomal pathway mediates autophagy levels, improves neuron survival rates, and alleviates depression and improves adverse psychiatric symptoms. Several studies[21 56-61] reported that the station post significantly improved blood flow, improved the inflammatory environment, and possibly enhanced immunity.

Ethics and dissemination

This study has been approved by the Ethics Committee of Shandong University of Traditional Chinese Medicine (Approval Number: 2021-025-KY). The results of this study will be published in a peer-reviewed journal, and trial participants will be informed via email or phone calls.

Footnotes

Author contributions

All authors contributed to the study design. HYB and XHL conceived the research idea, LLZ and XMX designed experiments, YXC, QW, XJQ, HHC, and LMW steered the ethics application, and GMC and WYS contributed to data analysis. JHJ, HS, XYJ, and HFL drafted the manuscript. All authors approved the final version of the manuscript.

Funding

This study was funded by the Science and Technology Project of Traditional Chinese Medicine of Shandong Province sponsored by Shandong Provincial Health Commission (Grant Number: 2020Z04). However, the sponsor was not involved in the conduct of the trial, date collection, and statistical processing. Final trial results and the publication of any reports are independent of the sponsor.

Consent for publication

Not required.

Competing interests

The authors declare none.

Table 1

Data collection schedule and visit assessments.

Measures	Baseline week	Intervention period (0–8 weeks)	End of intervention (9 weeks)	Follow-up period (9–16 weeks)	Follow-up (17 weeks)
Participant characteristics	√				
Mobile phone tremor test	√		√		√
CRST (A + B)	√		√		√

For peer review only - http://bmjopen.bmj.com/site/about/guidelines.xhtml

**CPET**	√		√		√
**UPDRS (II, III)**	√		√		√
**Hoehn-YaHr**	√		√		√
**Adverse events[1]**		√		√	
**Combined medication[2]**		√		√	

*[1]**Adverse events** : Adverse event at any visit during treatment sessions and over the 36 weeks will be monitored. The research team will review all trial protocols, monitor patient safety, and investigate any adverse events.*

*[2]**Combined medication:** Patients will be asked if they have used other medications during the treatment. If they have medications type and dose will be recorded. CRST (A+B): Clinical tremor Rating Scale; CPET: Cardiopulmonary exercise test; UPDRS (II, III): Unified Parkinson's Disease Rating Scale (II, III).*

For peer review only

Figure legend

Figures 1 and 2: Demonstration of Qigong movement of ZhanZhuang.

Figure 3: Test flow chart.

Table 1: Data collection schedule and visit assessments.

References

1. Cryan JF, O'Riordan KJ, Sandhu K, et al. The gut microbiome in neurological disorders. *The Lancet Neurology* 2020;19(2):179-94. doi: 10.1016/s1474-4422(19)30356-4 [published Online First: 2019/11/23]

2. Hayes MT. Parkinson's Disease and Parkinsonism. *The American journal of medicine* 2019;132(7):802-07. doi: 10.1016/j.amjmed.2019.03.001 [published Online First: 2019/03/21]

3. Meshack RP, Norman KE. A randomized controlled trial of the effects of weights on amplitude and frequency of postural hand tremor in people with Parkinson's disease. *Clinical rehabilitation* 2002;16(5):481-92. doi: 10.1191/0269215502cr521oa [published Online First: 2002/08/27]

4. Bian YJ, Wang YX, Zhou YQ. Research progress on brain network model of tremor

For peer review only - http://bmjopen.bmj.com/site/about/guidelines.xhtml

mechanism in Parkinson's disease . *China Comprehensive Clinical* 2016;32(8):749-53. doi: 10.3760/cma.j.issn.1008-6315.2016.08.023.

5. Heusinkveld LE, Hacker ML, Turchan M, et al. Impact of Tremor on Patients With Early Stage Parkinson's Disease. *Frontiers in neurology* 2018;9:628. doi: 10.3389/fneur.2018.00628 [published Online First: 2018/08/21]

6. Sarno M, Gaztanaga W, Banerjee N, et al. Revisiting eligibility for deep brain stimulation: Do preoperative mood symptoms predict outcomes in Parkinson's disease patients? *Parkinsonism & related disorders* 2019;63:131-36. doi: 10.1016/j.parkreldis.2019.02.019 [published Online First: 2019/02/26]

7. Armstrong MJ, Okun MS. Choosing a Parkinson Disease Treatment. *Jama* 2020;323(14):1420. doi: 10.1001/jama.2020.1224 [published Online First: 2020/04/15]

8. Cerri S, Blandini F. An update on the use of non-ergot dopamine agonists for the treatment of Parkinson's disease. *Expert opinion on pharmacotherapy* 2020;21(18):2279-91. doi: 10.1080/14656566.2020.1805432 [published Online First: 2020/08/18]

9. Liu HJ, He TZ, Zhang JL, et al. [Systematic review and screening of basic Chinese herbs for traditional Chinese medicine compounds combined with levodopa medicine in treatment of Parkinson's disease]. *Zhongguo Zhong yao za zhi = Zhongguo zhongyao zazhi = China journal of Chinese materia medica* 2020;45(24):6043-52. doi: 10.19540/j.cnki.cjcmm.20200717.501 [published Online First: 2021/01/27]

10. McGill A, Houston S, Lee RY. Dance for Parkinson's: a new framework for research on its physical, mental, emotional, and social benefits. *Complementary therapies in medicine* 2014;22(3):426-32. doi: 10.1016/j.ctim.2014.03.005 [published Online First:

For peer review only - http://bmjopen.bmj.com/site/about/guidelines.xhtml

For peer review only

2014/06/08]

11. Bond AE, Shah BB, Huss DS, et al. Safety and Efficacy of Focused Ultrasound Thalamotomy for Patients With Medication-Refractory, Tremor-Dominant Parkinson Disease: A Randomized Clinical Trial. *JAMA Neurol* 2017;74(12):1412-18. doi: 10.1001/jamaneurol.2017.3098 [published Online First: 2017/10/31]

12. Giordano M, Caccavella VM, Zaed I, et al. Comparison between deep brain stimulation and magnetic resonance-guided focused ultrasound in the treatment of essential tremor: a systematic review and pooled analysis of functional outcomes. *Journal of neurology, neurosurgery, and psychiatry* 2020;91(12):1270-78. doi: 10.1136/jnnp-2020-323216 [published Online First: 2020/10/16]

13. Kremer NI, Pauwels RWJ, Pozzi NG, et al. Deep Brain Stimulation for Tremor: Update on Long-Term Outcomes, Target Considerations and Future Directions. *Journal of clinical medicine* 2021;10(16) doi: 10.3390/jcm10163468 [published Online First: 2021/08/28]

14. Pooja NA, Pahuja SK, Veer K. Significance of MRI Guided Focused Ultrasound Thalamotomy for Parkinson's Disease: A Review. *Current medical imaging* 2021;17(6):714-19. doi: 10.2174/1573405616666201223142505 [published Online First: 2020/12/29]

15. Kadkhodaie M, Sharifnezhad A, Ebadi S, et al. Effect of eccentric-based rehabilitation on hand tremor intensity in Parkinson disease. *Neurological sciences : official journal of the Italian Neurological Society and of the Italian Society of Clinical Neurophysiology* 2020;41(3):637-43. doi: 10.1007/s10072-019-04106-9 [published Online First: 2019/11/19]

For peer review only - http://bmjopen.bmj.com/site/about/guidelines.xhtml

16. Cikajlo I, Peterlin Potisk K. Advantages of using 3D virtual reality based training in persons with Parkinson's disease: a parallel study. *Journal of neuroengineering and rehabilitation* 2019;16(1):119. doi: 10.1186/s12984-019-0601-1 [published Online First: 2019/10/19]

17. Duncan RP, Earhart GM. Randomized controlled trial of community-based dancing to modify disease progression in Parkinson disease. *Neurorehabilitation and neural repair* 2012;26(2):132-43. doi: 10.1177/1545968311421614 [published Online First: 2011/10/01]

18. King LK, Almeida QJ, Ahonen H. Short-term effects of vibration therapy on motor impairments in Parkinson's disease. *NeuroRehabilitation* 2009;25(4):297-306. doi: 10.3233/nre-2009-0528 [published Online First: 2009/12/29]

19. Farashi S, Kiani L, Bashirian S. Effect of Exercise on Parkinson's Disease Tremor: A Meta-analysis Study. *Tremor and other hyperkinetic movements (New York, NY)* 2021;11:15. doi: 10.5334/tohm.599 [published Online First: 2021/05/14]

20. Xu RD. ZhanZhaung, all-round tendon therapy. *HEALTH FOR THE ELDERLY AND MIDDLE-AGED* 2011(8):39.

21. Guo Y. Study on physiological and gene expression mechanism of "Regulating heart" effect of three-circle Zhanzhuang on alleviating depression [PhD]. Beijing University of Chinese Medicine, 2019.

22. Gao YM. Research on the special function and value of Taiji post in modern. *Wushu Martial arts research* . 2016;1(03):81-83.

23. Khuzema A, Brammatha A, Arul Selvan V. Effect of home-based Tai Chi, Yoga or

For peer review only

For peer review only - http://bmjopen.bmj.com/site/about/guidelines.xhtml

conventional balance exercise on functional balance and mobility among persons with idiopathic Parkinson's disease: An experimental study. *Hong Kong physiotherapy journal : official publication of the Hong Kong Physiotherapy Association Limited = Wu li chih liao* 2020;40(1):39-49. doi: 10.1142/s1013702520500055 [published Online First: 2020/06/04]

24. Li F, Harmer P. Economic Evaluation of a Tai Ji Quan Intervention to Reduce Falls in People With Parkinson Disease, Oregon, 2008-2011. *Preventing chronic disease* 2015;12:E120. doi: 10.5888/pcd12.140413 [published Online First: 2015/08/01]

25. Zhang YZ, Ma Z, Zheng MY, et al. Effect of Taijiquan on balance disorder in elderly Parkinson's disease and rheumatism related diseases. *Chinese Journal of Gerontology.* 2016;36(23):6031-33.

26. Zhang L, Jiang W F, Sun C H, et al. Progress of tai Chi exercise in the treatment of motor and non-motor symptoms in Parkinson's disease. *Neurology and Neurorehabilitation*. 2017;13(03):138-41.

27. Parkinson's disease and Movement Disorders Group, Neurology Branch of Chinese Medical Association, Parkinson's disease and Movement Disorders Group, Neurology Branch of Chinese Medical Doctor Association. Chinese guidelines for the treatment of Parkinson's disease (4th edition). *Chinese Journal of Neurology*. 2020;53(12):973-86.

28. Xu MY. Quantitative analysis and comparison of UPDRS and smart phones on upper limb tremor in PD patients [Master's degree]. Kunming Medical University, 2020.

29. ATS/ACCP Statement on cardiopulmonary exercise testing. *American journal of respiratory*

For peer review only - http://bmjopen.bmj.com/site/about/guidelines.xhtml

and critical care medicine 2003;167(2):211-77. doi: 10.1164/rccm.167.2.211 [published Online First: 2003/01/14]

30. Balady GJ, Arena R, Sietsema K, et al. Clinician's Guide to cardiopulmonary exercise testing in adults: a scientific statement from the American Heart Association. *Circulation* 2010;122(2):191-225. doi: 10.1161/CIR.0b013e3181e52e69 [published Online First: 2010/06/30]

31. Sun XG. ZhanZhuang requirements and difficulties of cardiopulmonary exercise test - data analysis diagrams and interpretation principles. *Chinese journal of Applied Physiology*. 2015;31(04):361-65+96-98.

32. Song Y. Clinical study of radial pulse wave in cardiopulmonary exercise test as the core of the overall management of chronic disease [Ms]. Chongqing Medical University, 2021.

33. Hansen JE, Sun XG, Stringer WW. A simple new visualization of exercise data discloses pathophysiology and severity of heart failure. *Journal of the American Heart Association* 2012;1(3):e001883. doi: 10.1161/jaha.112.001883 [published Online First: 2012/11/07]

34. Sun XG, Hansen JE, Beshai JF, et al. Oscillatory breathing and exercise gas exchange abnormalities prognosticate early mortality and morbidity in heart failure. *Journal of the American College of Cardiology* 2010;55(17):1814-23. doi: 10.1016/j.jacc.2009.10.075 [published Online First: 2010/04/24]

35. Sun XG. New 9 figure diagram of cardiopulmonary exercise test for strengthening cardiopulmonary metabolism and other overall functions. *Chinese journal of applied physiology*. 2015, 31 (4) : 369-373 + 399-400 DOI: 10.13459 / j.carol carroll nki cjap.

For peer review only - http://bmjopen.bmj.com/site/about/guidelines.xhtml

2015.04.018.

36. Beaver WL, Wasserman K, Whipp BJ. A new method for detecting anaerobic threshold by gas exchange. *Journal of applied physiology (Bethesda, Md : 1985)* 1986;60(6):2020-7. doi: 10.1152/jappl.1986.60.6.2020 [published Online First: 1986/06/01]

37. Kim Y, Lai B, Mehta T, et al. Exercise Training Guidelines for Multiple Sclerosis, Stroke, and Parkinson Disease: Rapid Review and Synthesis. *American journal of physical medicine & rehabilitation* 2019;98(7):613-21. doi: 10.1097/phm.0000000000001174 [published Online First: 2019/03/08]

38. Sun MS, Jin H, Sun X, et al. Free Radical Damage in Ischemia-Reperfusion Injury: An Obstacle in Acute Ischemic Stroke after Revascularization Therapy. *Oxidative medicine and cellular longevity* 2018;2018:3804979. doi: 10.1155/2018/3804979 [published Online First: 2018/05/18]

39. Chen Z, Zhong C. Oxidative stress in Alzheimer's disease. *Neuroscience bulletin* 2014;30(2):271-81. doi: 10.1007/s12264-013-1423-y [published Online First: 2014/03/26]

40. Oertel W, Schulz JB. Current and experimental treatments of Parkinson disease: A guide for neuroscientists. *Journal of neurochemistry* 2016;139 Suppl 1:325-37. doi: 10.1111/jnc.13750 [published Online First: 2016/09/01]

41. Chang KH, Chen CM. The Role of Oxidative Stress in Parkinson's Disease. *Antioxidants (Basel, Switzerland)* 2020;9(7) doi: 10.3390/antiox9070597 [published Online First: 2020/07/12]

42. Dias V, Junn E, Mouradian MM. The role of oxidative stress in Parkinson's disease. *Journal*

For peer review only - http://bmjopen.bmj.com/site/about/guidelines.xhtml

of Parkinson's disease 2013;3(4):461-91. doi: 10.3233/jpd-130230 [published Online First: 2013/11/21]

43. Smeyne M, Smeyne RJ. Glutathione metabolism and Parkinson's disease. *Free radical biology & medicine* 2013;62:13-25. doi: 10.1016/j.freeradbiomed.2013.05.001 [published Online First: 2013/05/15]

44. Liu YW, Liu WB, Feng XY, et al. Effects of station piling on superoxide dismutase in human erythrocytes. *Journal of Shaanxi University of Traditional Chinese Medicine.* 1991(01):27.

45. Yang J, Sadler TR, Givrad TK, et al. Changes in brain functional activation during resting and locomotor states after unilateral nigrostriatal damage in rats. *NeuroImage* 2007;36(3):755-73. doi: 10.1016/j.neuroimage.2007.03.010 [published Online First: 2007/05/08]

46. Petzinger GM, Fisher BE, McEwen S, et al. Exercise-enhanced neuroplasticity targeting motor and cognitive circuitry in Parkinson's disease. *The Lancet Neurology* 2013;12(7):716-26. doi: 10.1016/s1474-4422(13)70123-6 [published Online First: 2013/06/19]

47. Zach H, Dirkx MF, Pasman JW, et al. Cognitive Stress Reduces the Effect of Levodopa on Parkinson's Resting Tremor. *CNS neuroscience & therapeutics* 2017;23(3):209-15. doi: 10.1111/cns.12670 [published Online First: 2017/01/11]

48. Lee HJ, Lee WW, Kim SK, et al. Tremor frequency characteristics in Parkinson's disease under resting-state and stress-state conditions. *Journal of the neurological sciences* 2016;362:272-7. doi: 10.1016/j.jns.2016.01.058 [published Online First: 2016/03/06]

For peer review only - http://bmjopen.bmj.com/site/about/guidelines.xhtml

49. Wang JQ. Functional magnetic resonance study on the relationship between motor and cognition in different subtypes of Parkinson's disease [PhD]. Zhejiang University, 2018.

50. Shao Y, Le W. Recent advances and perspectives of metabolomics-based investigations in Parkinson's disease. *Molecular neurodegeneration* 2019;14(1):3. doi: 10.1186/s13024-018-0304-2 [published Online First: 2019/01/13]

51. Adjustment of three-circle ZhanZhuang on mental health level of medical college students. 30th anniversary of world Medical Qigong Association; 2019; Beijing, China.

52. Huang BS. Research on the interference of ZhanZhuang and Wuxing Music on College Students' attention [Master's degree]. Guangxi University for Nationalities, 2017.

53. The effect of exercise exercise on cognitive control (ERP Study). 2014 China Sports Physiology and Biochemistry Academic Conference; 2014; Guiyang, Guizhou Province, China.

54. Yang SH, Yang QF, Shi JM, et al. Observation of eeg spectrum changes in standing pile exercisers. CHINESE JOURNAL OF INTEGRATED TRADITIONAL AND WESTERN MEDICINE. 1994(11):643-46.

55. Fang C. Study on intervention of regimen pile on mild and moderate hypertension [Master's degree]. Beijing Sport University, 2014.

56. Ma JG. Experimental study on the brain mechanism of Yi Quan (Dacheng Quan) ZhangZhuang promoting emotion and Executive Control (NEAR Infrared Spectroscopy)

For peer review only

For peer review only - http://bmjopen.bmj.com/site/about/guidelines.xhtml

[Master. Capital Institute of Physical Education, 2011.

57. Li CR, Ren DP, Bai Zeng-hua, et al. Changes of integral emG in the initial stage of Taijiquan non-pole ZhanZhuang. *Practical Internal Medicine journal of Traditional Chinese Medicine*. 2013; 27 (08) : 13-14.

58. Liu Xl. A preliminary study on the Physiological Mechanism of Tai Chi ZhanZhuang. *FUJIAN SPORTS SCIENCE AND TECHNOLOGY*. 1996(4):20-21,37.

59. Wu D, XIONG KY, Song Y. Study on electromyographic changes of xingyiquan three-body potential station. *Journal of Beijing Sport University.* 2012;35(04):65-69+73.

60. Zhou XZ, Zhang ZX, Cui HJ, et al. Study on the changes of core muscle group surface electromyography by post work. *Journal of Liaoning University of Chinese Medicine.* 2018;20(11):66-69.

61. Yang SH, Yang QF, SHI JM, et al. Observation on the change of eeg frequency spectrum of exercise-ZhanZhaung exercisers. *Chinese Journal of Integrated Traditional and Western Medicine.* 1994(11):643-46.

For peer review only

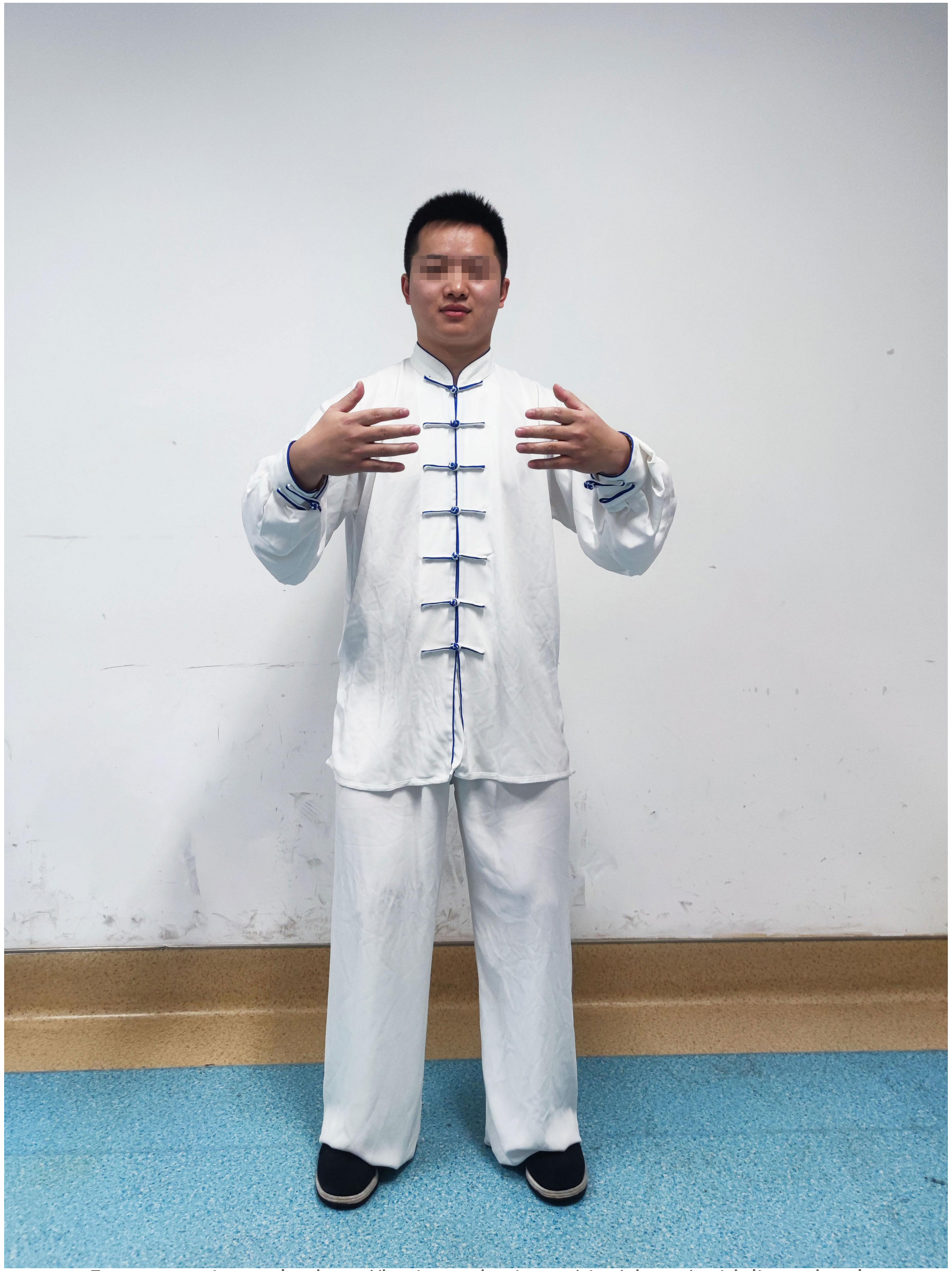

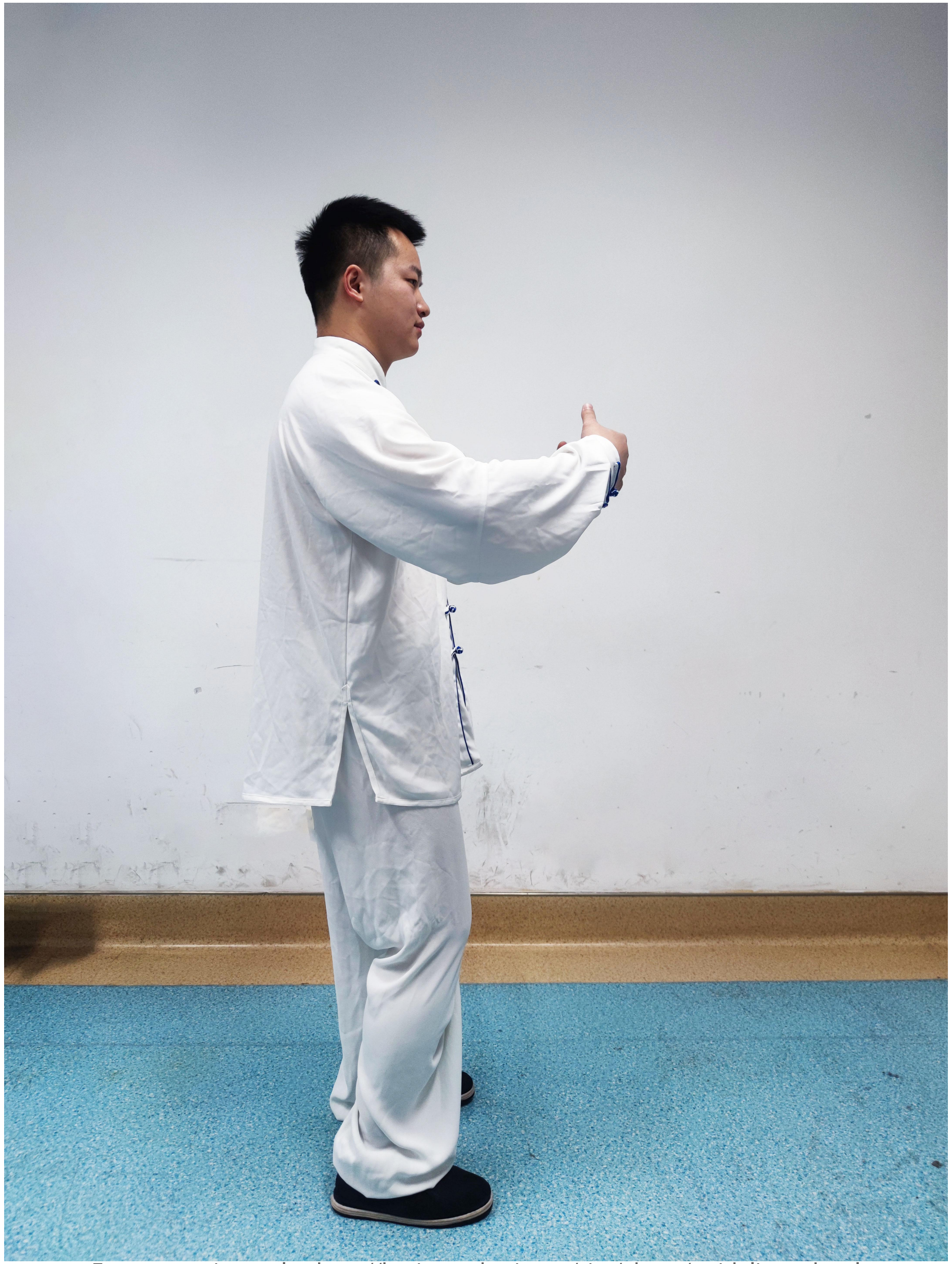

For peer review only - http://bmjopen.bmj.com/site/about/guidelines.xhtml

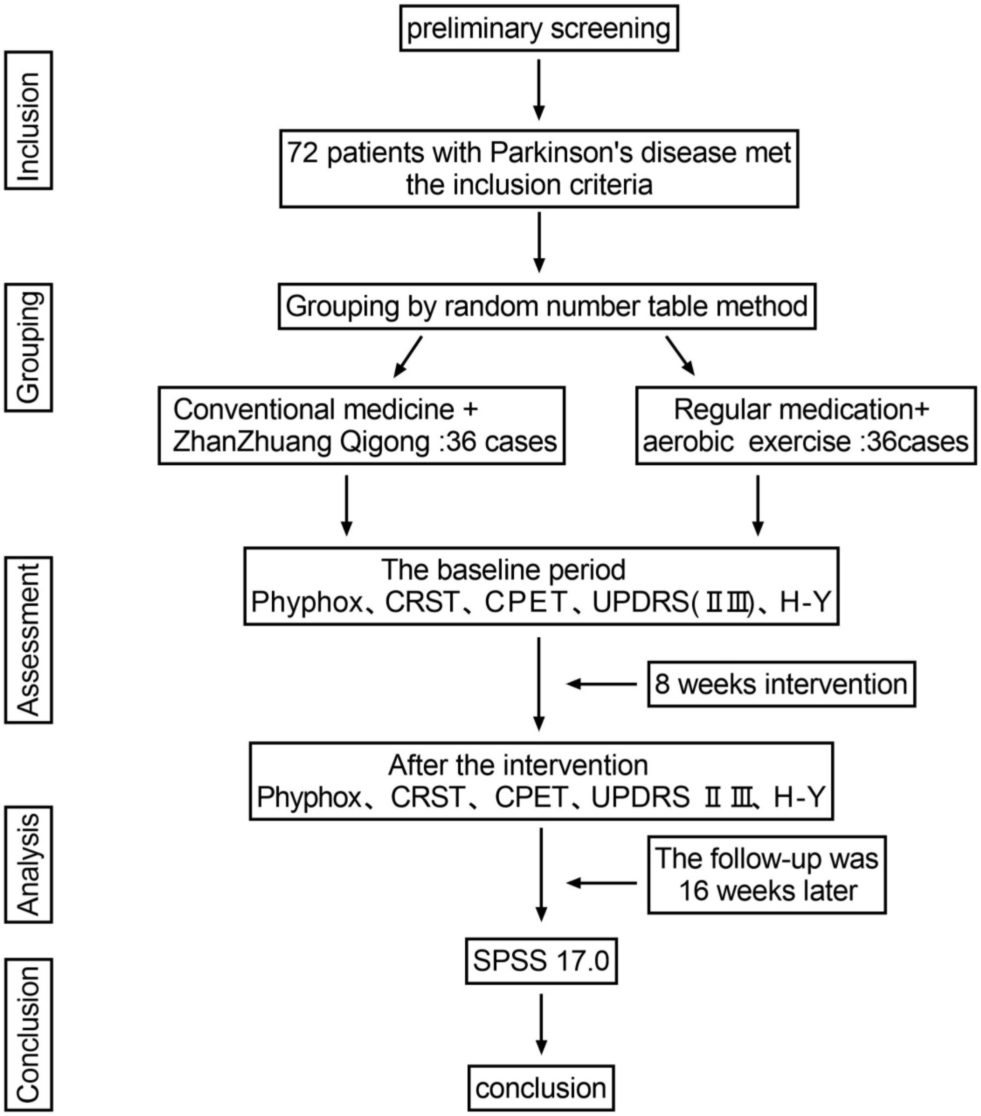

Figure 3

1417x1605mm (72 x 72 DPI)

ICMJE Presentation

**Date:**	3/8/2022
**Your name:**	Yuxiao Chen
**Manuscript Title:**	Effect of Zhan Zhuang Qigong on static tremor of the upper extremities and ability to exercise aerobic exercise in patients with mild to moderate Parkinson's disease: a study protocol of randomized controlled trials
**Manuscript number (if known):**	bmjopen-2021-059625. Type R1

For the sake of transparency, we ask that you disclose all relationships/activities/interests listed below in relation to the content of your manuscript. "Related" means any relationship with a for-profit or non-profit third party whose interests may be affected by the content of the Manuscript. Disclosure represents a commitment to transparency and does not necessarily indicate bias. If you're not sure if you want to list a relationship/activity/interest, it's best to do so.

The author's relationships/activities/interests should be broadly defined. For example, if your manuscript is related to the epidemiology of hypertension, you should declare all relationships with the manufacturer of an antihypertensive drug, even if the drug is not mentioned in the manuscript.

In item 1 below, all support for the work reported in this manuscript is reported, with no time limit. For all other projects, the time frame for disclosure is the last 36 months.

		Name all entities with which you have this relationship or indicate that there are no entities (add rows as needed)	Specifications/comments (e.g., whether to pay you or your institution)
		**Time frame: Since the initial planning of the work**	
**1**	All support for this manuscript (e.g., funding, provision of study materials, medical writing, essay processing fees, etc.) **There is no time limit for this project.**	☒ **No**	
			Click the Tab key to add additional rows.
		**Time frame: The last 36 months**	
**2**	Grants or contracts from any entity (if not specified in item 1 above).	☒ **No**	
**3**	Royalties or licenses	☒ **No**	

For peer review only - http://bmjopen.bmj.com/site/about/guidelines.xhtml

		Name all entities with which you have this relationship or indicate that there are no entities (add rows as needed)	Specifications/comments (e.g., whether to pay you or your institution)
**4**	Consulting fees	☒ **No**	
**5**	Payments or honorariums for lectures, lectures, speech bureaus, manuscript writing or educational activities	☒ **No**	
**6**	Payment of expert testimony	☒ **No**	
**7**	Support for meetings and/or trips	☒ **No**	
**8**	Planned, issued or pending patents	☒ **No**	
**9**	Participate in the Data Security Monitoring Committee or advisory committee	☒ **No**	
**10**	Leadership or trustee roles on other boards, societies, committees or advocacy groups, paid or unpaid	☐ **No**	

For peer review only - http://bmjopen.bmj.com/site/about/guidelines.xhtml

		Name all entities with which you have this relationship or indicate that there are no entities (add rows as needed)	Specifications/comments (e.g., whether to pay you or your institution)
**11**	Stocks or stock options	☒ **No**	
**12**	Receive equipment, medicines, medical treatment, medical writing, gifts, or other services	☒ **No**	
**13**	Other financial or non-financial benefits	☒ **No**	

Please indicate your consent by placing an "X" next to the following statement:

☒ I guarantee that I have answered every question and have not changed the wording of any question on this form.

For peer review only - http://bmjopen.bmj.com/site/about/guidelines.xhtml

I, the Submitting Author has the right to grant and does grant on behalf of all authors of the Work, an exclusive licence and for contributions from authors where BMJ has agreed a CC-BY licence shall apply, and/or ii) in accordance with the terms applicable for US Federal Government officers or employees acting as part of their official duties; on a worldwide, perpetual, irrevocable, royalty-free basis to BMJ Publishing Group Ltd ("BMJ") its licensees and where the relevant Journal is co-owned by BMJ to the co-owners of the Journal, to publish the Work in BMJ Open and any other BMJ products and to exploit all rights, as set out in our licence.

For peer review only

Reporting checklist for protocol of a clinical trial.

Based on the SPIRIT guidelines.

Instructions to authors

Complete this checklist by entering the page numbers from your manuscript where readers will find each of the items listed below.

Your article may not currently address all the items on the checklist. Please modify your text to include the missing information. If you are certain that an item does not apply, please write "n/a" and provide a short explanation.

Upload your completed checklist as an extra file when you submit to a journal.

In your methods section, say that you used the SPIRITreporting guidelines, and cite them as:

Chan A-W, Tetzlaff JM, Gøtzsche PC, Altman DG, Mann H, Berlin J, Dickersin K, Hróbjartsson A, Schulz KF, Parulekar WR, Krleža-Jerić K, Laupacis A, Moher D. SPIRIT 2013 Explanation and Elaboration: Guidance for protocols of clinical trials. BMJ. 2013;346:e7586

		Reporting Item	Page Number
**Administrative information**			
Title	#1	Descriptive title identifying the study design, population, interventions, and, if applicable, trial acronym	1
Trial registration	#2a	Trial identifier and registry name. If not yet registered,	1

For peer review only

		name of intended registry	
Trial registration: data set	#2b	All items from the World Health Organization Trial Registration Data Set	2
Protocol version	#3	Date and version identifier	2
Funding	#4	Sources and types of financial, material, and other support	10
Roles and responsibilities: contributorship	#5a	Names, affiliations, and roles of protocol contributors	10
Roles and responsibilities: sponsor contact information	#5b	Name and contact information for the trial sponsor	10
Roles and responsibilities: sponsor and funder	#5c	Role of study sponsor and funders, if any, in study design; collection, management, analysis, and interpretation of data; writing of the report; and the decision to submit the report for publication, including whether they will have ultimate authority over any of these activities	10
Roles and responsibilities: committees	#5d	Composition, roles, and responsibilities of the coordinating centre, steering committee, endpoint adjudication committee, data management team, and other individuals or groups overseeing the trial, if applicable (see Item 21a for data monitoring committee)	10

Introduction

For peer review only

Background and rationale	#6a	Description of research question and justification for undertaking the trial, including summary of relevant studies (published and unpublished) examining benefits and harms for each intervention	2-3
Background and rationale: choice of comparators	#6b	Explanation for choice of comparators	2-3
Objectives	#7	Specific objectives or hypotheses	1
Trial design	#8	Description of trial design including type of trial (eg, parallel group, crossover, factorial, single group), allocation ratio, and framework (eg, superiority, equivalence, non-inferiority, exploratory)	3
**Methods: Participants, interventions, and outcomes**			
Study setting	#9	Description of study settings (eg, community clinic, academic hospital) and list of countries where data will be collected. Reference to where list of study sites can be obtained	3
Eligibility criteria	#10	Inclusion and exclusion criteria for participants. If applicable, eligibility criteria for study centres and individuals who will perform the interventions (eg,	4

For peer review only

		surgeons, psychotherapists)	
Interventions: description	#11a	Interventions for each group with sufficient detail to allow replication, including how and when they will be administered	3
Interventions: modifications	#11b	Criteria for discontinuing or modifying allocated interventions for a given trial participant (eg, drug dose change in response to harms, participant request, or improving / worsening disease)	n/a
Interventions: adherance	#11c	Strategies to improve adherence to intervention protocols, and any procedures for monitoring adherence (eg, drug tablet return; laboratory tests)	3
Interventions: concomitant care	#11d	Relevant concomitant care and interventions that are permitted or prohibited during the trial	3
Outcomes	#12	Primary, secondary, and other outcomes, including the specific measurement variable (eg, systolic blood pressure), analysis metric (eg, change from baseline, final value, time to event), method of aggregation (eg, median, proportion), and time point for each outcome. Explanation of the clinical relevance of chosen efficacy and harm outcomes is strongly recommended	5-6
Participant timeline	#13	Time schedule of enrolment, interventions (including any run-ins and washouts), assessments, and visits for participants. A schematic diagram is highly recommended (see Figure)	10

For peer review only

Sample size	#14	Estimated number of participants needed to achieve study objectives and how it was determined, including clinical and statistical assumptions supporting any sample size calculations	5
Recruitment	#15	Strategies for achieving adequate participant enrolment to reach target sample size	3
**Methods: Assignment of interventions (for controlled trials)**			
Allocation: sequence generation	#16a	Method of generating the allocation sequence (eg, computer-generated random numbers), and list of any factors for stratification. To reduce predictability of a random sequence, details of any planned restriction (eg, blocking) should be provided in a separate document that is unavailable to those who enrol participants or assign interventions	3
Allocation concealment mechanism	#16b	Mechanism of implementing the allocation sequence (eg, central telephone; sequentially numbered, opaque, sealed envelopes), describing any steps to conceal the sequence until interventions are assigned	5
Allocation: implementation	#16c	Who will generate the allocation sequence, who will enrol participants, and who will assign participants to interventions	5

For peer review only

Blinding (masking)	#17a	Who will be blinded after assignment to interventions (eg, trial participants, care providers, outcome assessors, data analysts), and how	5
Blinding (masking): emergency unblinding	#17b	If blinded, circumstances under which unblinding is permissible, and procedure for revealing a participant's allocated intervention during the trial	5
**Methods: Data collection, management, and analysis**			
Data collection plan	#18a	Plans for assessment and collection of outcome, baseline, and other trial data, including any related processes to promote data quality (eg, duplicate measurements, training of assessors) and a description of study instruments (eg, questionnaires, laboratory tests) along with their reliability and validity, if known. Reference to where data collection forms can be found, if not in the protocol	6-7
Data collection plan: retention	#18b	Plans to promote participant retention and complete follow-up, including list of any outcome data to be collected for participants who discontinue or deviate from intervention protocols	6-7
Data management	#19	Plans for data entry, coding, security, and storage, including any related processes to promote data quality (eg, double data entry; range checks for data values).	6-7

For peer review only

		Reference to where details of data management procedures can be found, if not in the protocol	
Statistics: outcomes	#20a	Statistical methods for analysing primary and secondary outcomes. Reference to where other details of the statistical analysis plan can be found, if not in the protocol	6-7
Statistics: additional analyses	#20b	Methods for any additional analyses (eg, subgroup and adjusted analyses)	6-7
Statistics: analysis population and missing data	#20c	Definition of analysis population relating to protocol non-adherence (eg, as randomised analysis), and any statistical methods to handle missing data (eg, multiple imputation)	6-7
**Methods: Monitoring**			
Data monitoring: formal committee	#21a	Composition of data monitoring committee (DMC); summary of its role and reporting structure; statement of whether it is independent from the sponsor and competing interests; and reference to where further details about its charter can be found, if not in the protocol. Alternatively, an explanation of why a DMC is not needed	6
Data monitoring: interim analysis	#21b	Description of any interim analyses and stopping guidelines, including who will have access to these interim results and make the final decision to terminate the trial	6
Harms	#22	Plans for collecting, assessing, reporting, and managing solicited and spontaneously reported adverse events and other unintended effects of trial interventions or trial	6

For peer review only

		conduct	
Auditing	#23	Frequency and procedures for auditing trial conduct, if any, and whether the process will be independent from investigators and the sponsor	6
**Ethics and dissemination**			
Research ethics approval	#24	Plans for seeking research ethics committee / institutional review board (REC / IRB) approval	1
Protocol amendments	#25	Plans for communicating important protocol modifications (eg, changes to eligibility criteria, outcomes, analyses) to relevant parties (eg, investigators, REC / IRBs, trial participants, trial registries, journals, regulators)	1
Consent or assent	#26a	Who will obtain informed consent or assent from potential trial participants or authorised surrogates, and how (see Item 32)	1
Consent or assent: ancillary studies	#26b	Additional consent provisions for collection and use of participant data and biological specimens in ancillary studies, if applicable	n/a
Confidentiality	#27	How personal information about potential and enrolled participants will be collected, shared, and maintained in order to protect confidentiality before, during, and after the trial	9-10
Declaration of	#28	Financial and other competing interests for principal	10

For peer review only

interests		investigators for the overall trial and each study site	
Data access	#29	Statement of who will have access to the final trial dataset, and disclosure of contractual agreements that limit such access for investigators	10
Ancillary and post trial care	#30	Provisions, if any, for ancillary and post-trial care, and for compensation to those who suffer harm from trial participation	n/a
Dissemination policy: trial results	#31a	Plans for investigators and sponsor to communicate trial results to participants, healthcare professionals, the public, and other relevant groups (eg, via publication, reporting in results databases, or other data sharing arrangements), including any publication restrictions	10
Dissemination policy: authorship	#31b	Authorship eligibility guidelines and any intended use of professional writers	10
Dissemination policy: reproducible research	#31c	Plans, if any, for granting public access to the full protocol, participant-level dataset, and statistical code	10
**Appendices**			
Informed consent materials	#32	Model consent form and other related documentation given to participants and authorised surrogates	n/a
Biological specimens	#33	Plans for collection, laboratory evaluation, and storage of biological specimens for genetic or molecular analysis in the current trial and for future use in ancillary studies, if applicable	n/a

For peer review only

None The SPIRIT Explanation and Elaboration paper is distributed under the terms of the Creative Commons Attribution License CC-BY-NC. This checklist can be completed online using https://www.goodreports.org/, a tool made by the EQUATOR Network in collaboration with Penelope.ai

For peer review only

BMJ Open

Effect of ZhanZhuang Qigong on upper limb static tremor and aerobic exercise capacity in patients with mild to moderate Parkinson's disease: Study Protocol for a Randomized controlled Trial

Journal:	*BMJ Open*
Manuscript ID	bmjopen-2021-059625.R2
Article Type:	Protocol
Date Submitted by the Author:	25-Jun-2022
Complete List of Authors:	zhang, linlin; Shandong University of Traditional Chinese Medicine,<br>Liu, Xihua; Shandong University of Traditional Chinese Medicine Affiliated Hospital<br>Xi, Xiaoming; Shandong University of Traditional Chinese Medicine<br>Chen, Yuxiao; Shandong University of Traditional Chinese Medicine<br>Wang, Qing; Shandong University of Traditional Chinese Medicine<br>Qu, Xinjie; Shandong University of Traditional Chinese Medicine<br>Cao, Haihao; Shandong University of Traditional Chinese Medicine<br>Wang, Limin; Shandong University of Traditional Chinese Medicine<br>Sun, Wenyu; Shandong University of Traditional Chinese Medicine Affiliated Hospital<br>Chen, Guoming; Shandong University of Traditional Chinese Medicine Affiliated Hospital<br>Liu, Huifen; Shandong University of Traditional Chinese Medicine<br>Jiang, Xiaoyu; Shandong University of Traditional Chinese Medicine<br>Su, Hang; Shandong University of Traditional Chinese Medicine<br>Jiang, Jiahui; Shandong University of Traditional Chinese Medicine<br>Bi, Hongyan; Shandong University of Traditional Chinese Medicine Affiliated Hospital
**Primary Subject Heading**:	Geriatric medicine
Secondary Subject Heading:	Rehabilitation medicine, Neurology
Keywords:	Parkinson-s disease < NEUROLOGY, GERIATRIC MEDICINE, Rehabilitation medicine < INTERNAL MEDICINE, Clinical trials < THERAPEUTICS

SCHOLARONE™ Manuscripts

Effect of ZhanZhuang Qigong on upper limb static tremor and aerobic exercise capacity in patients with mild to moderate Parkinson's disease: Study Protocol for a Randomized controlled Trial

Linlin Zhang, MD[1]; Xihua Liu, Ph. D[2]; Xiaoming Xi, MD[1]; Yuxiao Chen, MD[1]; Qing Wang, MD[1]; Xinjie Qu, MD[1]; Haihao Cao[1], MD[1]; Limin Wang[1], MD[1]; Wenyu Sun, MD[2]; Guoming Chen, MD[2]; Huifen Liu, MD[1]; Xiaoyu Jiang, MD[1]; Hang Su, MD[1]; Jiahui Jiang, MD[1]; Hongyan Bi, MD[1].

1: Shandong University of Traditional Chinese Medicine, Jinan, China

2: Shandong University of Traditional Chinese Medicine Affiliated Hospital, Jinan, China

Correspondence

Hongyan Bi, Shandong University of Traditional Chinese Medicine Affiliated Hospital, Jinan, China

Tel: 18800460276

Email: Hy__bi@163.com

Abstract

Introduction: Currently, the first choice for the clinical treatment of static tremor in Parkinson's disease (PD) is drug therapy, however side effects are common. In recent years, the effects of physical therapy on PD has become a serious research focus. Studies have indicated that aerobic and resistance exercises alleviate PD movement disorders and improve aerobic capacity, but the effects of Qigong on PD static tremor and aerobic capacity remain unknown.

Methods and analysis

Objective: To observe the effects of Zhanzhuang Qigong on upper limb static tremor and aerobic capacity in patients with PD, we established a rigorous randomized, parallel-controlled, assignment hidden, evaluator-blinded protocol. **Methods**: Seventy-two patients with PD, at the Affiliated Hospital of Shandong University of Traditional Chinese Medicine, were recruited and randomly divided into a control (n = 36) and experimental group (n = 36). The intervention group received conventional medicine plus ZhanZhuang Qigong exercises 5 times a week at 30 min each time, over an 8 week period. The long-term effects of ZhanZhuang Qigong on PD was investigated after the intervention. Phyphox APP, CRST, CPET, UPDRS（II, III) were used to evaluate tremor, aerobic capacity， and motor function ingroups. **Discussion**: we are investigating the effects of ZhanZhuang Qigong on upper limb static tremor and aerobic capacity in PD patients. If positive are identified, they will add a new research direction and evidence for the clinical exploration of exercise therapy for PD.

Ethics and dissemination

This study was approved by the Ethics Committee of Shandong University of Traditional Chinese Medicine (Approval Number: 2021-025-KY). The Committee will be informed of any changes to the trial protocol, such as intervention intensity, outcome indicators and data collection. Study results will be presented as a paper at an international

For peer review only - http://bmjopen.bmj.com/site/about/guidelines.xhtml

conference or in a journal.

Test registration Number

The study protocol was registered in the Chinese Clinical Trial Registry (Registration number: ChiCTR2100053529)

Version:

20211124

Key words: ZhanZhuang Qigong, Parkinson's disease, tremor, aerobic exercise capacity

Strengths and limitations of this study

1. Currently, the clinical effects of physical therapy toward Parkinson's tremor are not ideal, with no clinical studies conducted on the effects of Qigong on Parkinson's tremor.
2. The mobile phone tremor test and cardiopulmonary exercise test used in our outcome assessments were clinically verified as accurate and reliable.
3. Our rigorous hidden allocation, randomized parallel controlled trial design is more reliable and accurate than pre-and post-trial controls previously used in PD clinical trials.
4. Staff turnover during the study intervention period may lead to bias in the final results.
5. we are unable to investigate other forms of Parkinson's tremor other than static tremor.

Introduction

Parkinson's disease (PD) is a degenerative disease of the central nervous system common in middle-aged and elderly individual[1]. The PD prevalence in individuals aged 65-69 is approximately 0.5%-1%, and in individuals over 80, 1-3%. With increased population aging, it is anticipated that PD prevalence and incidence will increase by more than 30% by 2030, and place tremendous pressure on families and society[2].

As one of the main motor symptoms of PD, static tremor is characterized by rhythmic and clay ball-like tremor and is accompanied by a large amplitude and frequency of 4-6Hz, which occurs more when the weight of affected limbs or other body structures is supported. The tremor is aggravated when general exercises (such as walking) or pressures increases, but is alleviated or disappears when the body moves at will. The tremor gradually spreads from the distal end of one limb to the other[3, 4]. Parkinson's tremor is one of the most troubling symptoms in patients with PD, which not only reduces daily living activities, but also imbues a sense of inferiority, depression, and reduces participation in society[5]. Currently, the most effective clinical treatment for PD remains drug therapy[2, 6, 7]. Levodopa is considered the best treatment compound for supplementing exogenous dopamine and controlling PD, but its long-term use gradually reduces its efficacy and leads to central and peripheral adverse reactions[8-10]. Moreover, levodopa elicits poor responses to PD static tremor and has no effect on non-motor symptoms[11]. Invasive surgical treatments, such as focused thalamotomy and deep brain stimulation provide significant tremor relief in the short term, but do not prevent neurodegenerative progression in underlying PD[8, 12-14]. Additionally, surgery is risky and may be accompanied by complications[12].

For peer review only - http://bmjopen.bmj.com/site/about/guidelines.xhtml

In recent years, PD symptom improvement by exercise therapy has gained considerable research traction. Several studies reported that aerobic and resistance exercises alleviated PD motor symptoms, including tremor[15-19]. Among traditional Chinese Qigong therapies, Tai Chi, Wuqinxi and Baduanjin are believed to enhance balance in PD patients and correct abnormal gait. However, few studies have focused on PD tremor and aerobic capacity[20].

ZhanZhuang Qigong is a basic technique in Tai Chi, and is a static, moderate, and low intensity aerobic exercise combining body and mind[21, 22]. Several clinical studies have reported the benefits of Tai Chi for PD motor and non-motor functions[23], Tai Chi is a low cost exercise, it reduces falls, improves posture control, walking ability, improves depression and insomnia symptoms, and improve quality of life. As a basic Tai Chi skill, the ZhangZhuang has many Tai Chi characteristics[24]. For example, ZhanZhuang movements combine body and mind, they control limb and trunk stability and change the center of gravity with the mind. Additionally, like Tai Chi, ZhanZhuang is also a closed circular motion technique which maintains action continuity and accuracy via adjustment, feedback, re-adjustment, and re-feedback, which improve the body's recognition of stimuli and improves involuntary tremor[25, 26].

Methods and analysis

The study is a two-arm, randomized, parallel-controlled trial with assignment-hiding and evaluator blindness. The main objective is to investigate the effects of ZhanZhuang on upper limb static tremor and aerobic capacity in patients with mild to moderate PD, so as to provide a safe, effective, low-cost, and easy-to-master aerobic exercise therapy for PD patients.

Patient and public involvement

The public is not involved in the study design. However, we will adjust intervention intensity and other details based on how participants respond to the intervention.

Study design and setting

The study will be conducted in the Affiliated Hospital of Shandong University of Traditional Chinese Medicine. We will include 72 patients with PD with obvious upper limb static tremor. Recruitment will begin on June 1, 2022 and run until June 1, 2023. Using the random number table method, 72 patients will be randomly divided into an intervention and control group in a 1:1 ratio. Basic patient information, including gender, age, disease course, education level, residence status and drug-use will be collected. Tremor and motor function tests will be performed at least 8 hours after patients stop taking medication.

The control group will be treated with conventional medicine and routine aerobics. According to the Chinese Guidelines for the Treatment of PD (4th edition), walking will be selected as the aerobic exercise mode in this group[27], five times a week at 30 min each time for 8 weeks. A combined cardiopulmonary exercise test (CPET) (treadmill test) will be used to measure peak oxygen uptake (peakVO_2) in PD patients at baseline and after intervention, and also anaerobic threshold (AT) and maximum heart rate (HRmax). During training, patients exercise intensity will be controlled at moderate exercise intensity

For peer review only - http://bmjopen.bmj.com/site/about/guidelines.xhtml

(HRmax (60%-80%) measured by CPET or 11-13 Rating of Perceived Exertion (RPE). Changes in peakVO_2 and AT at baseline and after intervention will be evaluated for improvements in aerobic exercise capacity. Walking will be performed about 1 after patients take their medication, and the tremor symptoms were resolved or disappeared.

The intervention group will take conventional medicine combined with ZhanZhuang Qigong. An experienced Qigong master will guide ZhanZhuang exercise. Week 1 will comprise the learning phase, while weeks 2-8 will be the practice phases, to be performed 5 times a week at 30 min each time. Intensity HRmax (60%-80%) will be measured using a cardiopulmonary combined exercise test or RPE test grade 11-13. Patients will be issued a training instruction manual and a personal exercise calendar to record the number of weekly exercises and time of each exercise. ZhanZhuang training will be about 1 h after patients take their medication, and tremor symptoms were relieved or disappeared. One auxiliary staff member will oversee safety measures.

The main points of Qigong movement of ZhanZhuang

Individuals should stand naturally, with feet about 30 cm apart, and parallel to the front. Arms around, with naturally open hands and two fingertips about 15 cm apart, Flex the knee hip about 5-10° and tilt the hip forward (Figures 1, 2).

Participants and Recruitment

Using the Affiliated Hospital of Shandong University of Traditional Chinese Medicine as the base, we will visit and recruit patients from the rehabilitation and neurology departments, and post recruitment advertisements on Wechat.

We will assess upper limb static tremor according to the Unified Parkinson's Disease Rating Scale (UPDRS) Part III and the Hoehn-Yahr classification (stages 1-3) (see Figure 3).

Inclusion criteria

(1) Primary PD agrees with recommended Chinese Clinical Diagnostic Criteria for PD (2016);
(2) 50-70 years old;
(3) Improved Hoehn-Yahr stage 1-3;
(4) Stable condition, taking Madopa, and standing for the "prescribed period";
(5) No serious cognitive dysfunction, has the ability to learn ZhanZhuang movements; (Mini-Mental State Examination (MMSE) score, illiteracy > 17, primary school > 20 and middle school > 22);
(6) Agrees to participate, and sign an informed consent sheet.

Exclusion criteria

(1) Secondary Parkinson's syndrome and Parkinson's superposition syndrome caused by cerebrovascular disease, drugs, and other causes;
(2) Patients with serious heart, lung, liver, kidney dysfunction or cancer;
(3) Those individuals who cannot stand due to serious knee diseases, e.g., severe knee

For peer review only - http://bmjopen.bmj.com/site/about/guidelines.xhtml

osteoarthritis and knee trauma;

(4) Patients with dementia or serious mental illness;

(5) Patients unable to stand or walk independently due to fracture, surgery, or other reasons;

(6) No tremors;

(7) The "opening period" tremor is obvious after taking medication.

Termination/shedding criteria

(1) Patients not cooperating with prescribed exercises or exercising poor compliance;

(2) Patients whose condition deteriorates or complications arise during the study should not continue.

Sample size

This study is designed as a randomized controlled trial. The main outcome is the mean relief of upper limb static tremor after treatment. A review of previous studies showed that aerobic exercise could reduce upper limb static tremor amplitude by approximately 60%. It is estimated the average tremor amplitude of the control group is approximately 76 ± 136 mm, and the intervention group is expected to be reduced by 50 mm after training. The test level α is set at 0.05 (bilateral), the degree of certainty 1-β is set at 0.80, and the control: intervention group ratio = 1:1. A sample size of 63 was calculated using *http://powerandsamplesize.com*. However, considering an abscission rate of about 15%, 72 patients with PD will be included.

Randomization and Allocation Concealment and Blinding

This study is single-blinded. Because of the Qigong intervention, training personnel and subjects are not blinded, however testers, data collectors, data scientists, and analysts are blinded. Implementation of training, evaluation and statistical analysis of the separation of the way, that is, the training personnel do not contact the test personnel. Before testing, the intervention and control groups were renamed as groups A and B, respectively. The test tester is only responsible for the test by number, and does not tell the group of the test subject. Data collection and processing personnel are separated; data collection personnel obtain group A or group B date based on the number and prove this information to the data statistician for statistical analysis. To ensure study objectivity, quality control personnel will identify groups after statistics are completed.

Assessment result

Primary outcome

(1) Static tremor of the upper limbs

① Mobile phone movement exercise test

Phyphox APP[28] is based on the Android system (version: 1. 1. 9). It collects the static tremor date of the upper limb of patients with an obvious tremor, at a frequency of 100Hz over 30 s.

Data collection on the static tremor: The patient relaxes, sits in a chair, places his/her hands on the armrest, and holds the mobile phone with the screen up on the shaking side.

For peer review only - http://bmjopen.bmj.com/site/about/guidelines.xhtml

At the same time, patients perform numerical subtraction calculations to distract their attention. The time from the beginning of the tremor is recorded over 30 s.

② The Clinical Tremor Rating Scale (CRST) A+B, CRST-A is used to evaluate the tremor at rest, postural, and intentional; CRST-B assesses handwriting (dominant hand only), wide and narrow spiral drawing, straight lines, and casting. For the dominant hand, the maximum sub-score of hand tremor is 32 for CRST (A+B) and 28 for the non-dominant hand. The higher the score, the more severe the tremor.

(2) Aerobic capacity

Combined Cardiopulmonary Exercise Test (CPET)

The cardiovit CS-200 cardiopulmonary combined exercise test system (SCHILLERA, Switzerland) is used for CPET. The treadmill test is performed, and the increasing rate set at 10W/min. Based on standards from Harbor-UCLA Center Cardiopulmonary Exercise Laboratory, USA, the resting state lung function examination, including fast lung capacity, slow lung capacity, maximum minute ventilation capacity, and diffusion function, should be completed first. Symptom limiting maximum CPET is then completed using continuous ramping regimen[29-31]. CPET is divided into four stages, including 3 min of rest, 3 min warm-up (no treadmill power), then exercise at an increasing power rate of 10W/min until symptom limiting maximum exercise, followed by more than 5min of recovery[32]. Continuous 12-lead ECG, non-invasive cuff blood pressure, and oxygen saturation measurements are required during the CPET process. PeakVO$_2$, AT, and HRmax are measured and recorded.

Secondary indicator

Motor symptoms and daily living abilities

(1) UPDRS IIandIII: The scale is divided into four parts. The first part evaluates the patient's mental behaviors and emotions. Part II assesses the patient's ability to live a daily life. Part III assesses the patient's motor functions; The total score in part Ⅳ reflects patient complications. We selected PartsIIand part III to evaluate the daily living activities and motor functions of patients, respectively.

(2) Revised Hoehn-Yahr Grading Scale: The scale is divided into eight stages, from 0-5 (0, 1, 1.5, 2, 2.5, 3, 4 and 5), to evaluate disease severity.

Adverse events (AEs)

AEs occurring during the study are recorded using a Case Report Form. For this trial, an AE will be defined as adverse and unexpected signs, symptoms or diseases associated with the intervention, such as falls, joint injuries, hypertension, etc. Participants will be asked about their medical history and any AEs they have experienced before training begins, and AEs will be recorded after each intervention. The principal investigator will be notified within 24 h if a serious adverse event (SAE) occurs. If subjects withdraw from the trial due to any AE, the study team will follow-up accordingly or until the AE is resolved, This information will be recorded in the original file. The principal investigator manages all

For peer review only - http://bmjopen.bmj.com/site/about/guidelines.xhtml

safety reports. Any AEs relating to the intervention will be reported to the Ethics Review Committee of the Affiliated Hospital of Shandong University of Chinese Medicine.

Statistical analysis

We will evaluate the outcome and collect data at week 9 of the intervention and follow up at week 17 of the intervention. (Table 1) . Basic patient information, mobile phone exercise test data, CRST scores, and UPDRS (II , III) scores will be exported to our computer system CSV format.

SPSS17.0 statistical software will be used for data processing and analysis: 1). Measurement data will be processed using the mean standard deviation ($x^2 \pm s$); 2). T tests will be used assess: normal distribution and homogeneity of the overall variance in both samples; 3). T tests will be used to assess: normal distributions, but uneven overall variance; 4). The Wilcoxon rank sum test will assess if date does not conform to a normal distribution; $P < 0.05$ indicates statistical significance.

Mobile exercise test data

The original form is the acceleration a (m/s) of three axes (X, Y, and Z axes) vector direction respectively), rotation radian w (rad/s). The MATLAB (version R 2018a, MathWorks, Inc, USA) computer program will be used to perform Fourier transformation of the data, and acceleration and rotation radian measurements by smart phone will be converted to corresponding frequency, peak amplitude, and speed date. Final phone test data will be presented as the amplitude and frequency of static tremors. Then, normal distribution tests will be conducted. Since the sample size is small ($N < 5000$), the Shapiro-Wilk normal test will be used. $P > 0.05$ indicates a normal distribution, while $P < 0.05$ indicates a non-normal distribution.

According to normal distribution test date, if mobile phone exercise test data is normally distributed and the variance uniform, mobile phone exercise test data in PD patients in different groups will be tested using with two sample independent T tests. $P < 0.05$ will indicated if the exercise is statistically significant between groups. If the data are not normally distributed, non-parametric tests (median and Mann-Whitney U tests) will be performed for comparison. $P < 0.05$ in median tests will indicate if differences in central tendency scores between groups are statistically significant.

CPET test data

After subjects complete the CPET test, raw date will be saved. According to standardized principles of CPET data analysis, and the interpretation of FDA clinical trials in the Harbor-UCLA Medical Center[33, 34], original breath-by-breath data from subjects will be segmsed every second from the CPET system software. Then, average the data for 10s and export the average data[31, 35]. The warm-up value is the average of the last 30s of the warm-up period, the anaerobic threshold value is the data value of 10s when the anaerobic threshold, and the peak value is the average of 30s during the extreme exercise. Anaerobic threshold analysis is performed using a v-slope method, i.e., the mean value of 10s of oxygen uptake when the CO_2 emission rate is significantly accelerated relative to oxygen uptake rate during movement[36].

For peer review only - http://bmjopen.bmj.com/site/about/guidelines.xhtml

Discussion

PD not only reduces patients quality of life, but also generates a heavy economic burden for society and families. Upper limb static tremor is one of the main PD motor symptoms. Dyskinesia may can also lead to decreased activity and aerobic capacity in PD patients. Currently, non-drug therapy for PD mainly includes aerobic exercise and resistance training. Previous studies reported those methods were beneficial for tremor, bradykinesia, balance, and quality of life in PD patients[37]. However, the effect of Qigong on PD static tremor and aerobic ability remains unknown.

Free radicals (OH)[38] are a strong oxides that induce lipid peroxidation in unsaturated fatty acids and produce lipid peroxidation (LPO), which damages proteins and DNA and lead to cell degeneration and death. Oxidative stress (OS)[39] represents an imbalanced state between oxidation and antioxidant effects in the body, which are important factors contributing to aging and disease. Due to increased activity of B-monoamine oxidase (MAO-B) in PD patients, excessive free radicals oxidize and destroy cell membranes[40]. At the same time, dopamine oxidation products in substantia nigra cells polymerize to form neuromelanin, which combines with iron to produce Fenton reactions and free radicals. Under normal circumstances, antioxidant molecules in brain cells are plentiful and include, glutathione (GSH), glutathione peroxidase (GSH-PX), and superoxide dismutase (SOD), thus free radicals generated by dopamine oxidation will not lead to oxidative stress[41, 42]. In PD, the substantia nigra is vulnerable to oxidative stress due to decreased ferritin and glutathione leves, and increased lipid peroxide, and iron ion concentrations[41-43]. Previous studies[11, 44] reported that ZhanZhuang improved SOD the activity levels in the body, prevented harm from superoxide free radicals, and exerted positive roles fighting aging, inflammation, and immune disease, Therefore, the ZhangZhuang may protect the substantia nigra in PD patients by increasing SOD activity levels, reducing substantia nigra lesions, slowing down PD progression, and improving overall symptoms in PD patients.

Currently, the generally accepted PD tremor mechanism in clinical practice is the induced-switch-regulation model proposed by Duval[4].

Induced: Pathological activity in the basal nucleus striatum induces rhythmic cluster discharge activity in thalamic neurons via he excessive input of inhibitory signals to thalamic cells, and changes the discharge mode.

Switch: Oscillations in internal and external networks of the thalamus combine and cause oscillations to continuously integrate and strengthen until they reach tremor production threshold values.

Regulation: After PD tremor-related oscillations are introduced to the cerebellum, it processes oscillation as random movements and adjusts tremors to ensure stability in oscillation activities, such as frequency and amplitude. Tremors transmitted to the cerebral cortex are then transmitted to the periphery via the cortical-spinal cord pathway in the form of oscillations, and are finally manifested body tremors. Yang[45], based on data from basal ganglia injury mice and rats models, reported that exercise training reduced over-activation of basal ganglia cortical circuits, thereby weakening tremor signal transmission, and alleviating tremors. Recent studies also reported these animal models were applicable to humans[46]. Mona[15] observed that upper limb centrifugal exercises effectively

For peer review only - http://bmjopen.bmj.com/site/about/guidelines.xhtml

reduced upper limb tremor amplitude in PD disease patients. ZhanZhuang as an aerobic exercise may also reduce tremors at the motor level.

PD tremor is influenced by attention and stress levels[47]. Previous studies reported that tremors were intentionally attenuated when attention was focused on them, and that PD tremors were is amplified by stress[48]. Jiaqiu Wang[49] using nuclear magnetic resonance (NMR) to analyze tremor subtypes in patients with PD, observed that the brains of tremor subtypes in patients with a wide range of functional connection between the frontal lobe and cerebellum, and UPDRS score III movement and the cognitive function score also existed significant correlation, that movement function and cognitive function in patients with PD has a high degree of correlation. DA substitution therapy also alleviated some motor characteristics in PD, but the observed beneficial effects on cognitive function were small[50]. Several studies[21, 51, 52] reported that ZhanZhuang enhanced the attention and cognitive ability of adolescents and the elderly. Yiyue Chen[53] observed that ZhanZhuang exercises increased P3 wave amplitude in frontal, central, and top regions, with a shorter incubation period when compared with baseline. P3 wave amplitude reflected the allocation of neural electrical resources, and latency which was related to the speed of stimulus classification. These results suggest that station post affected neural electrical processing related to cognitive control , by changing neural electrical resource allocation and stimulus classification speeds, thereby affecting cognitive function. Therefore, the station post may indirectly improve tremor performance by enhancing cognitive ability in PD patients.

Executive control is a general control mechanism where individuals coordinate different cognitive processes and resources to ensure their cognitive system implements specific goals in a flexible and optimized way when completing complex cognitive tasks. Executive control also coordinates brain commands and muscle movements. Sihuan Yang[54] conducted a 1 year ZhanZhuang exercise in healthy college students, and observed that EEG activity coefficients in students who participated in ZhanZhuang exercises were generally decreased, and that EEG's showed a high synchronization trend. Whole brain ordering was improved, and brain activities were more coordinated and synchronized. ChaoFang[55] reported that ZhanZhuang can exercised the muscles of the whole body statically, provided benign stimulation to the brain, and adjusted the disorder of excitement and inhibition of the cerebral cortex. Jianguo Ma[56] also confirmed that ZhanZhuang improved subject response speeds and accuracy, and also improved the executive control levels in the brain. ZhanZhuang combines body and mind, it focuses on controlling the body with the mind, and mobilizing muscle groups in the trunk and limbs to participate in this process[21]. This body and mind combination stimulates the nervous system and increase its sensitivity, ensuring more efficient and precise muscle control. Additionally, ZhanZhuangs places the body in a stress state of postural control for long period, with previous clinical studies showing that stress significantly affects neural plasticity[56]. Thus, higher levels of executive control and finer, more efficient postural control may benefit PD tremor relief.

YU Guo[21] observed that B cell receptor and NF-κB signaling pathways were

For peer review only - http://bmjopen.bmj.com/site/about/guidelines.xhtml

significantly enriched in college students after ZhanZhuang, suggesting the technique may enhance in vivo immunity and improve inflammatory microenvironments by activating B cell receptor signaling and improving T lymphocyte levels, or regulating NF-κB signaling. Regulating valine, leucine, and isoleucine metabolism, prevents excessive glutamate accumulation, increases astrocyte and 5-hydroxytryptamine levels, and prevents neuronal necrosis and apoptosis. The specific regulation of the lysosomal pathway mediates autophagy levels, improves neuron survival rates, and alleviates depression and improves adverse psychiatric symptoms. Several studies[21, 56-61] reported that the station post significantly improved blood flow, improved the inflammatory environment, and possibly enhanced immunity.

Ethics and dissemination

This study has been approved by the Ethics Committee of Shandong University of Traditional Chinese Medicine (Approval Number: 2021-025-KY). The results of this study will be published in a peer-reviewed journal, and trial participants will be informed via email or phone calls.

Footnotes

Author contributions

All authors contributed to the study design. HYB and XHL conceived the research idea, LLZ and XMX designed experiments, YXC, QW, XJQ, HHC, and LMW steered the ethics application, and GMC and WYS contributed to data analysis. JHJ, HS, XYJ, and HFL drafted the manuscript. All authors approved the final version of the manuscript.

Funding

This study was funded by the Science and Technology Project of Traditional Chinese Medicine of Shandong Province sponsored by Shandong Provincial Health Commission (Grant Number: 2020Z04). However, the sponsor was not involved in the conduct of the trial, date collection, and statistical processing. Final trial results and the publication of any reports are independent of the sponsor.

Consent for publication

Not required.

Competing interests

The authors declare none.

Table 1

Data collection schedule and visit assessments.

Measures	Baseline week	Intervention period (0–8 weeks)	End of intervention (9 weeks)	Follow-up period (9–16 weeks)	Follow-up (17 weeks)
Participant characteristics	√				

For peer review only

For peer review only - http://bmjopen.bmj.com/site/about/guidelines.xhtml

**Mobile phone tremor test**	√		√		√
**CRST (A + B)**	√		√		√
**CPET**	√		√		√
**UPDRS (II, III)**	√		√		√
**Hoehn-YaHr**	√		√		√
**Adverse events[1]**		√		√	
**Combined medication[2]**		√		√	

*[1]**Adverse events** : Adverse event at any visit during treatment sessions and over the 36 weeks will be monitored. The research team will review all trial protocols, monitor patient safety, and investigate any adverse events.*

*[2]**Combined medication:** Patients will be asked if they have used other medications during the treatment. If they have medications type and dose will be recorded. CRST (A+B): Clinical tremor Rating Scale; CPET: Cardiopulmonary exercise test; UPDRS (II, III): Unified Parkinson's Disease Rating Scale (II, III).*

Figure legend

Figures 1 and 2: Demonstration of Qigong movement of ZhanZhuang.

Figure 3: Test flow chart.

Table 1: Data collection schedule and visit assessments.

References

1. Cryan JF, O'Riordan KJ, Sandhu K, et al. The gut microbiome in neurological disorders. *The Lancet Neurology* 2020;19(2):179-94. doi: 10.1016/s1474-4422(19)30356-4 [published Online First: 2019/11/23]

2. Hayes MT. Parkinson's Disease and Parkinsonism. *The American journal of medicine* 2019;132(7):802-07. doi: 10.1016/j.amjmed.2019.03.001 [published Online First: 2019/03/21]

3. Meshack RP, Norman KE. A randomized controlled trial of the effects of weights on amplitude and frequency of postural hand tremor in people with Parkinson's disease. *Clinical rehabilitation* 2002;16(5):481-92. doi: 10.1191/0269215502cr521oa [published Online

For peer review only

For peer review only - http://bmjopen.bmj.com/site/about/guidelines.xhtml

First: 2002/08/27]

4. Bian YJ, Wang YX, Zhou YQ, et al. Advances in brain network modeling of tremor mechanism in Parkinson's disease. China Comprehensive Clinical. 2016;32(8):749-53. doi: 10.3760/cma.j.issn.1008-6315.2016.08.023

5. Heusinkveld LE, Hacker ML, Turchan M, et al. Impact of Tremor on Patients With Early Stage Parkinson's Disease. *Frontiers in neurology* 2018;9:628. doi: 10.3389/fneur.2018.00628 [published Online First: 2018/08/21]

6. Sarno M, Gaztanaga W, Banerjee N, et al. Revisiting eligibility for deep brain stimulation: Do preoperative mood symptoms predict outcomes in Parkinson's disease patients? *Parkinsonism & related disorders* 2019;63:131-36. doi: 10.1016/j.parkreldis.2019.02.019 [published Online First: 2019/02/26]

7. Armstrong MJ, Okun MS. Choosing a Parkinson Disease Treatment. *Jama* 2020;323(14):1420. doi: 10.1001/jama.2020.1224 [published Online First: 2020/04/15]

8. Cerri S, Blandini F. An update on the use of non-ergot dopamine agonists for the treatment of Parkinson's disease. *Expert opinion on pharmacotherapy* 2020;21(18):2279-91. doi: 10.1080/14656566.2020.1805432 [published Online First: 2020/08/18]

9. Liu HJ, He TZ, Zhang JL, et al. [Systematic review and screening of basic Chinese herbs for traditional Chinese medicine compounds combined with levodopa medicine in treatment of Parkinson's disease]. *Zhongguo Zhong yao za zhi = Zhongguo zhongyao zazhi = China journal of Chinese materia medica* 2020;45(24):6043-52. doi: 10.19540/j.cnki.cjcmm.20200717.501 [published Online First: 2021/01/27]

10. McGill A, Houston S, Lee RY. Dance for Parkinson's: a new framework for research on its

For peer review only - http://bmjopen.bmj.com/site/about/guidelines.xhtml

physical, mental, emotional, and social benefits. *Complementary therapies in medicine* 2014;22(3):426-32. doi: 10.1016/j.ctim.2014.03.005 [published Online First: 2014/06/08]

11. Bond AE, Shah BB, Huss DS, et al. Safety and Efficacy of Focused Ultrasound Thalamotomy for Patients With Medication-Refractory, Tremor-Dominant Parkinson Disease: A Randomized Clinical Trial. *JAMA Neurol* 2017;74(12):1412-18. doi: 10.1001/jamaneurol.2017.3098 [published Online First: 2017/10/31]
12. Giordano M, Caccavella VM, Zaed I, et al. Comparison between deep brain stimulation and magnetic resonance-guided focused ultrasound in the treatment of essential tremor: a systematic review and pooled analysis of functional outcomes. *Journal of neurology, neurosurgery, and psychiatry* 2020;91(12):1270-78. doi: 10.1136/jnnp-2020-323216 [published Online First: 2020/10/16]
13. Kremer NI, Pauwels RWJ, Pozzi NG, et al. Deep Brain Stimulation for Tremor: Update on Long-Term Outcomes, Target Considerations and Future Directions. *Journal of clinical medicine* 2021;10(16) doi: 10.3390/jcm10163468 [published Online First: 2021/08/28]
14. Pooja NA, Pahuja SK, Veer K. Significance of MRI Guided Focused Ultrasound Thalamotomy for Parkinson's Disease: A Review. *Current medical imaging* 2021;17(6):714-19. doi: 10.2174/1573405616666201223142505 [published Online First: 2020/12/29]
15. Kadkhodaie M, Sharifnezhad A, Ebadi S, et al. Effect of eccentric-based rehabilitation on hand tremor intensity in Parkinson disease. *Neurological sciences : official journal of the Italian Neurological Society and of the Italian Society of Clinical Neurophysiology*

For peer review only - http://bmjopen.bmj.com/site/about/guidelines.xhtml

2020;41(3):637-43. doi: 10.1007/s10072-019-04106-9 [published Online First: 2019/11/19]

16. Cikajlo I, Peterlin Potisk K. Advantages of using 3D virtual reality based training in persons with Parkinson's disease: a parallel study. *Journal of neuroengineering and rehabilitation* 2019;16(1):119. doi: 10.1186/s12984-019-0601-1 [published Online First: 2019/10/19]

17. Duncan RP, Earhart GM. Randomized controlled trial of community-based dancing to modify disease progression in Parkinson disease. *Neurorehabilitation and neural repair* 2012;26(2):132-43. doi: 10.1177/1545968311421614 [published Online First: 2011/10/01]

18. King LK, Almeida QJ, Ahonen H. Short-term effects of vibration therapy on motor impairments in Parkinson's disease. *NeuroRehabilitation* 2009;25(4):297-306. doi: 10.3233/nre-2009-0528 [published Online First: 2009/12/29]

19. Farashi S, Kiani L, Bashirian S. Effect of Exercise on Parkinson's Disease Tremor: A Meta-analysis Study. *Tremor and other hyperkinetic movements (New York, NY)* 2021;11:15. doi: 10.5334/tohm.599 [published Online First: 2021/05/14]

20. Moon S, Sarmento CVM, Smirnova IV, et al. Effects of Qigong Exercise on Non-Motor Symptoms and Inflammatory Status in Parkinson's Disease: A Protocol for a Randomized Controlled Trial. Medicines (Basel) 2019;6(1) doi: 10.3390/medicines6010013 [published Online First: 2019/01/24]

21. Guo Y. Study on the physiological and gene expression mechanism of "regulating heart" effect of three-circle Zhanzhuang on alleviating depression [doctor]. Beijing University

For peer review only - http://bmjopen.bmj.com/site/about/guidelines.xhtml

of Chinese Medicine, 2019.

22. Gao YM. Gao Yongmei. Gao Yongmei. Research on the special function and value of Taiji Zhanzhuang in modern Wushu. Martial Arts Studies .2016;1(03):81-83.

23. Khuzema A, Brammatha A, Arul Selvan V. Effect of home-based Tai Chi, Yoga or conventional balance exercise on functional balance and mobility among persons with idiopathic Parkinson's disease: An experimental study. *Hong Kong physiotherapy journal : official publication of the Hong Kong Physiotherapy Association Limited = Wu li chih liao* 2020;40(1):39-49. doi: 10.1142/s1013702520500055 [published Online First: 2020/06/04]

24. Li F, Harmer P. Economic Evaluation of a Tai Ji Quan Intervention to Reduce Falls in People With Parkinson Disease, Oregon, 2008-2011. *Preventing chronic disease* 2015;12:E120. doi: 10.5888/pcd12.140413 [published Online First: 2015/08/01]

25. Zhang YZ, Ma Z, Zheng MY, et al. Effect of Taijiquan on balance disorder of senile Parkinson's disease and rheumatism related diseases . Chinese Journal of Gerontology. 2016;36(23):6031-33.

26. Zhang L, Jiang WF, Sun CH, et al. Progress in Tai Chi Quan for treatment of moto ir and non-motor symptoms of Parkinson's disease. Journal of Neurology and Neurorehabilitation. 2017;13(03):138-41

27. Parkinson's disease and Movement Disorders Group, Neurology Branch of Chinese Medical Association, Parkinson's disease and Movement Disorders Group, Neurology Branch of Chinese Medical Doctor Association. Guidelines for the Treatment of Parkinson's disease in China (4th edition). Chinese Journal of Neurology.

For peer review only - http://bmjopen.bmj.com/site/about/guidelines.xhtml

2020;53(12):973-86.

28. Xu MY. Quantitative assessment of Parkinsonian hand tremor based on the UPDRS scale and smartphone [Master]. Kunming Medical University, 2020.

29. ATS/ACCP Statement on cardiopulmonary exercise testing. *American journal of respiratory and critical care medicine* 2003;167(2):211-77. doi: 10.1164/rccm.167.2.211 [published Online First: 2003/01/14]

30. Balady GJ, Arena R, Sietsema K, et al. Clinician's Guide to cardiopulmonary exercise testing in adults: a scientific statement from the American Heart Association. *Circulation* 2010;122(2):191-225. doi: 10.1161/CIR.0b013e3181e52e69 [published Online First: 2010/06/30]

31. Sun XG. Standardizing clinical performance,data analysis,graphics display,interpretation and report for cardiopulmonary exercise testing. Chinese Journal of Applied Physiology. 2015;31(04):361-65+96-98.

32. Song Y. Clinical Studies On radial artery pulse Waves in the over all management of Chronic Disease With Cardiopulmonary. [Master]. Chongqing Medical University, 2021.

33. Hansen JE, Sun XG, Stringer WW. A simple new visualization of exercise data discloses pathophysiology and severity of heart failure. *Journal of the American Heart Association* 2012;1(3):e001883. doi: 10.1161/jaha.112.001883 [published Online First: 2012/11/07]

34. Sun XG, Hansen JE, Beshai JF, et al. Oscillatory breathing and exercise gas exchange abnormalities prognosticate early mortality and morbidity in heart failure. *Journal of the American College of Cardiology* 2010;55(17):1814-23. doi: 10.1016/j.jacc.2009.10.075

For peer review only

For peer review only - http://bmjopen.bmj.com/site/about/guidelines.xhtml

[published Online First: 2010/04/24]

35. Sun XG. The new 9 panels display of data from cardiopulmonary exercise test,emphasizing holistic integrative multi-systemic function. Chinese Journal of Applied Physiology. 2015;31(04):369-73+99-400.

36. Beaver WL, Wasserman K, Whipp BJ. A new method for detecting anaerobic threshold by gas exchange. *Journal of applied physiology (Bethesda, Md : 1985)* 1986;60(6):2020-7. doi: 10.1152/jappl.1986.60.6.2020 [published Online First: 1986/06/01]

37. Kim Y, Lai B, Mehta T, et al. Exercise Training Guidelines for Multiple Sclerosis, Stroke, and Parkinson Disease: Rapid Review and Synthesis. *American journal of physical medicine & rehabilitation* 2019;98(7):613-21. doi: 10.1097/phm.0000000000001174 [published Online First: 2019/03/08]

38. Sun MS, Jin H, Sun X, et al. Free Radical Damage in Ischemia-Reperfusion Injury: An Obstacle in Acute Ischemic Stroke after Revascularization Therapy. *Oxidative medicine and cellular longevity* 2018;2018:3804979. doi: 10.1155/2018/3804979 [published Online First: 2018/05/18]

39. Chen Z, Zhong C. Oxidative stress in Alzheimer's disease. *Neuroscience bulletin* 2014;30(2):271-81. doi: 10.1007/s12264-013-1423-y [published Online First: 2014/03/26]

40. Oertel W, Schulz JB. Current and experimental treatments of Parkinson disease: A guide for neuroscientists. *Journal of neurochemistry* 2016;139 Suppl 1:325-37. doi: 10.1111/jnc.13750 [published Online First: 2016/09/01]

41. Chang KH, Chen CM. The Role of Oxidative Stress in Parkinson's Disease. *Antioxidants*

For peer review only - http://bmjopen.bmj.com/site/about/guidelines.xhtml

(Basel, Switzerland) 2020;9(7) doi: 10.3390/antiox9070597 [published Online First: 2020/07/12]

42. Dias V, Junn E, Mouradian MM. The role of oxidative stress in Parkinson's disease. *Journal of Parkinson's disease* 2013;3(4):461-91. doi: 10.3233/jpd-130230 [published Online First: 2013/11/21]

43. Smeyne M, Smeyne RJ. Glutathione metabolism and Parkinson's disease. *Free radical biology & medicine* 2013;62:13-25. doi: 10.1016/j.freeradbiomed.2013.05.001 [published Online First: 2013/05/15]

44. Liu YW, Liu WB, Feng XY, et al. Effect of station post work on superoxide dismutase in human erythrocytes. Journal of Shaanxi College of Traditional Chinese Medicine. 1991(01):27.

45. Yang J, Sadler TR, Givrad TK, et al. Changes in brain functional activation during resting and locomotor states after unilateral nigrostriatal damage in rats. *NeuroImage* 2007;36(3):755-73. doi: 10.1016/j.neuroimage.2007.03.010 [published Online First: 2007/05/08]

46. Petzinger GM, Fisher BE, McEwen S, et al. Exercise-enhanced neuroplasticity targeting motor and cognitive circuitry in Parkinson's disease. *The Lancet Neurology* 2013;12(7):716-26. doi: 10.1016/s1474-4422(13)70123-6 [published Online First: 2013/06/19]

47. Zach H, Dirkx MF, Pasman JW, et al. Cognitive Stress Reduces the Effect of Levodopa on Parkinson's Resting Tremor. *CNS neuroscience & therapeutics* 2017;23(3):209-15. doi: 10.1111/cns.12670 [published Online First: 2017/01/11]

For peer review only - http://bmjopen.bmj.com/site/about/guidelines.xhtml

48. Lee HJ, Lee WW, Kim SK, et al. Tremor frequency characteristics in Parkinson's disease under resting-state and stress-state conditions. *Journal of the neurological sciences* 2016;362:272-7. doi: 10.1016/j.jns.2016.01.058 [published Online First: 2016/03/06]

49. Wang JQ. Investigation of the Relationship between Motor andCognition of Different Subtypes of Parkinson’s Disease by Functional Magnetic Resonance Imaging [Doctor]. Zhejiang University.2018.

50. Shao Y, Le W. Recent advances and perspectives of metabolomics-based investigations in Parkinson's disease. *Molecular neurodegeneration* 2019;14(1):3. doi: 10.1186/s13024-018-0304-2 [published Online First: 2019/01/13]

51. Adjustment of three-circle Zhanzhuang on mental health level of medical college students. 30th anniversary of world Medical Qigong Association; 2019; Beijing China.

52. Huang BS. A study on the station and five elements of music intervetion to college students' attention.[Master]. Guangxi University for Nationalities, 2017.

53. The effect of exercise exercise on cognitive control (ERP Study). 2014 China Sports Physiology and Biochemistry Academic Conference; 2014; Guiyang, Guizhou Province, China..

54. Yang SH, Yang QF, Shi JM, et al. Observation on the change of eeg frequency spectrum of exercisers. Chinese Journal of Integrated Traditional and Western Medicine. 1994(11):643-46.

55. Fang C. Study on intervention of regimen pile on mild and moderate hypertension [Master]. Beijing Sport University, 2014..

56. Ma JG. Experimental Study on the Brain Mechanism of Yi Quan (Da Cheng Quan) Station

For peer review only - http://bmjopen.bmj.com/site/about/guidelines.xhtml

Post promoting Emotion and Executive Control (NEAR Infrared Spectroscopy) [Master]. Capital Institute of Physical Education, 2011.

57. Li CR, Ren DP, Bai ZH, et al. Variation of Surface Electromyography at The Initial Stage of Wuji Stance. Journal of Practical Traditional Chinese Internal Medicine. 2013;27(08):13-14.

58. Liu XL. Preliminary study on physiological mechanism of Taiji station pile. Fujian Sports Science and Technology. 1996(4):20-21,37.

59. Wu D, Xiong KY, Song Y. Electromyography Analysis of Santishi of Ying Yie. Journal of beijing university of physical education. 2012;35(04):65-69+73.

60. Zhou XZ, Zhang ZX, Cui HJ, et al. Study on the Change of Surface Electromyography Variation of Core Muscles by Standing Qigong. Journal of Liaoning University of Traditional Chinese Medicine. 2018;20(11):66-69.

61. Yang SH, Yang QF, SHI JM, et al. Observation on the change of eeg frequency spectrum of exercisers. Chinese Journal of Integrated Traditional and Western Medicine. 1994(11):643-46.

For peer review only - http://bmjopen.bmj.com/site/about/guidelines.xhtml

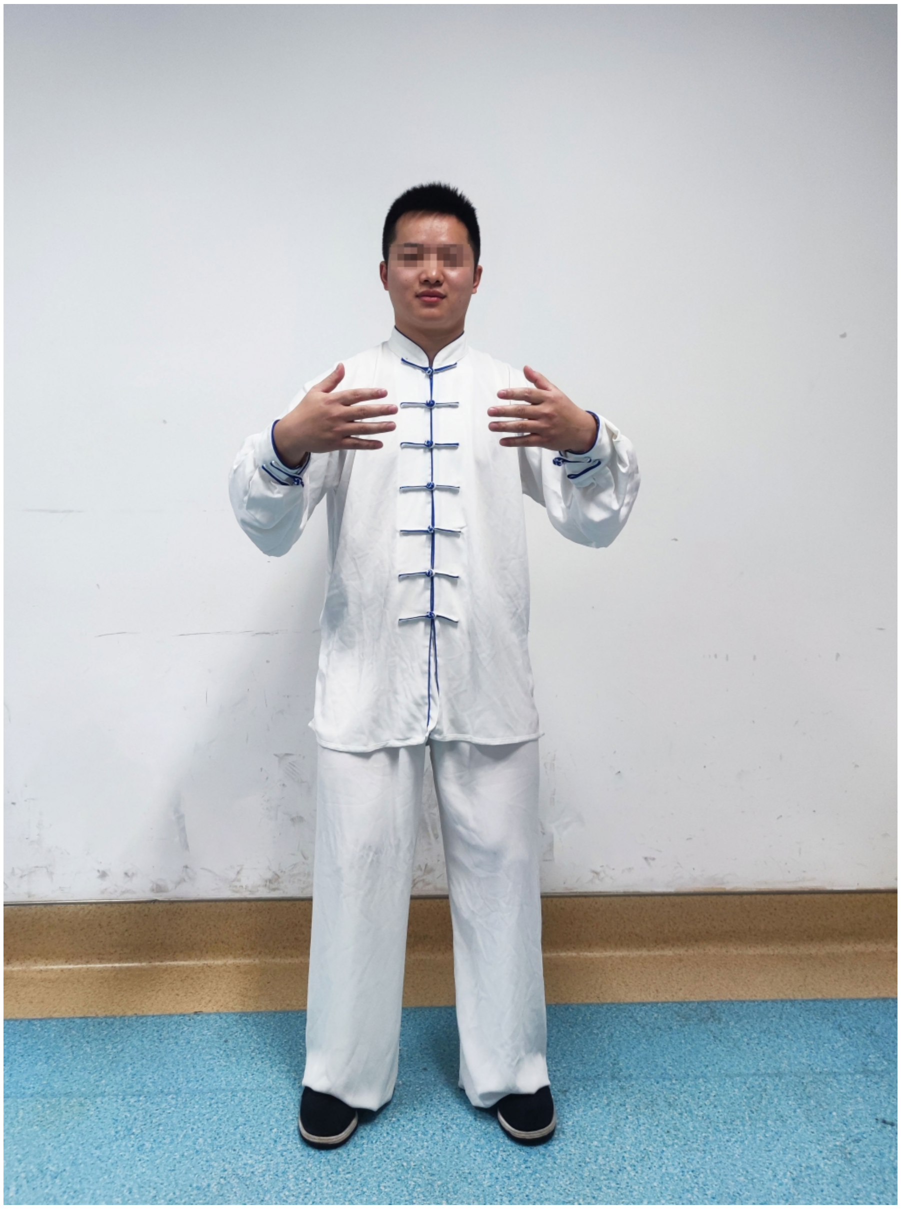

Figure 1

1248x1675mm (72 x 72 DPI)

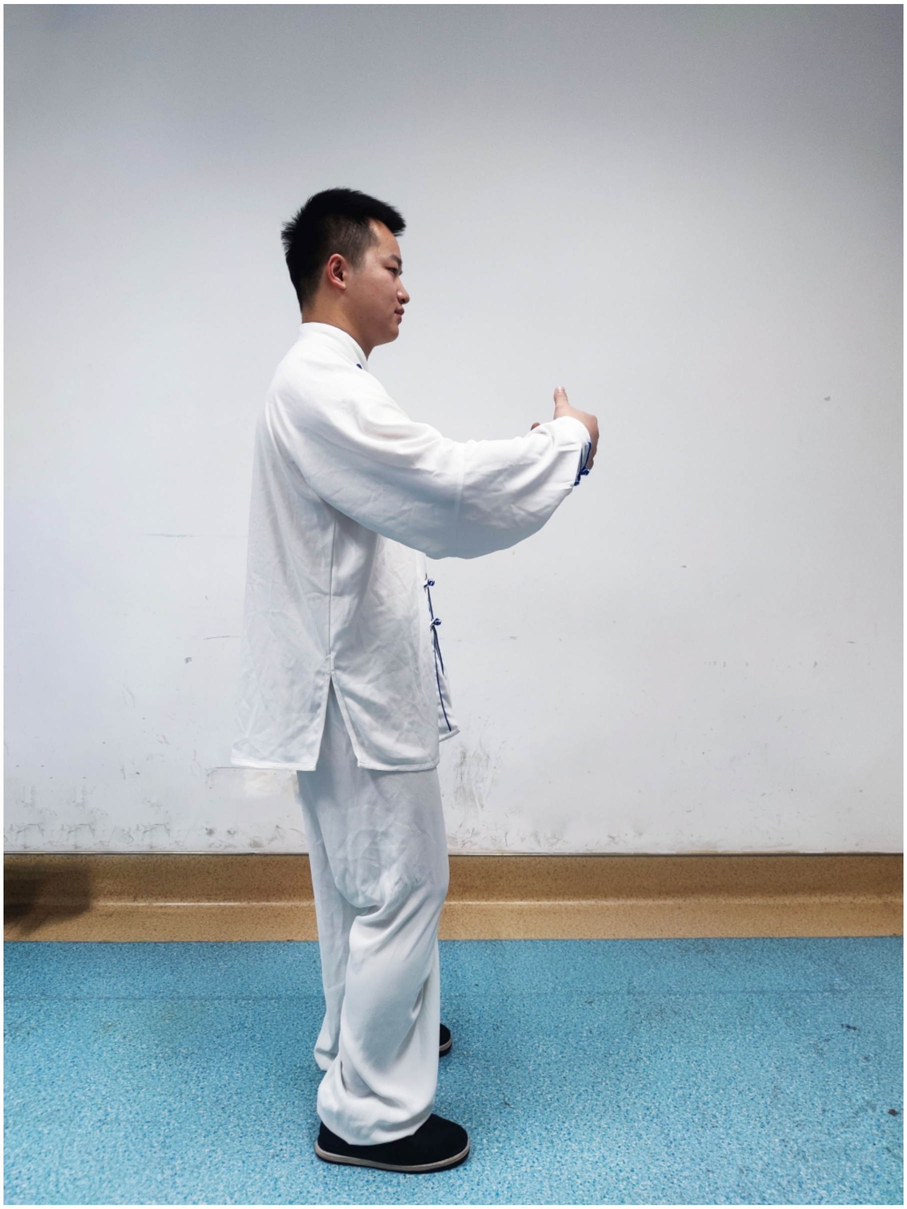

Figure 2

1532x2043mm (72 x 72 DPI)

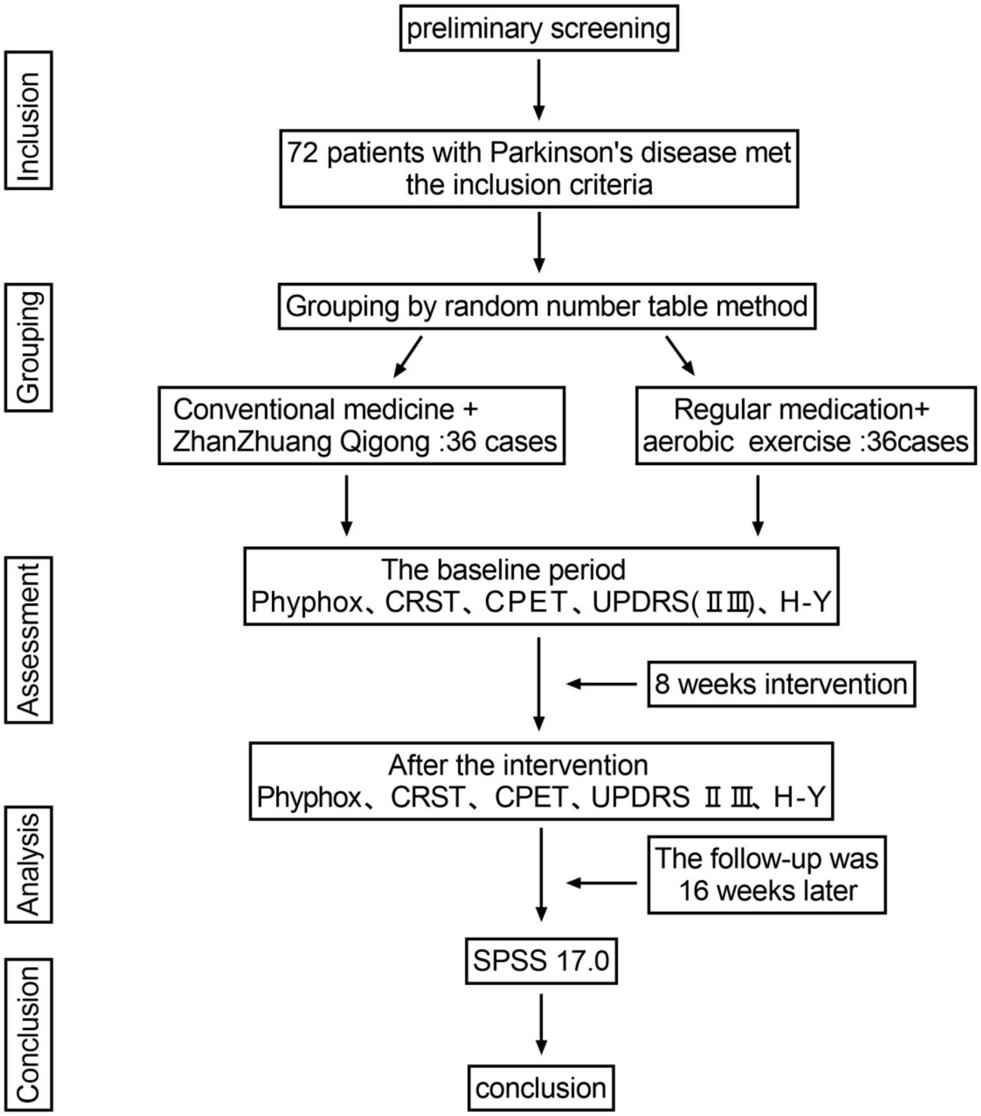

Figure 3

1417x1605mm (72 x 72 DPI)

Reporting checklist for protocol of a clinical trial.

Based on the SPIRIT guidelines.

Instructions to authors

Complete this checklist by entering the page numbers from your manuscript where readers will find each of the items listed below.

Your article may not currently address all the items on the checklist. Please modify your text to include the missing information. If you are certain that an item does not apply, please write "n/a" and provide a short explanation.

Upload your completed checklist as an extra file when you submit to a journal.

In your methods section, say that you used the SPIRITreporting guidelines, and cite them as:

Chan A-W, Tetzlaff JM, Gøtzsche PC, Altman DG, Mann H, Berlin J, Dickersin K, Hróbjartsson A, Schulz KF, Parulekar WR, Krleža-Jerić K, Laupacis A, Moher D. SPIRIT 2013 Explanation and Elaboration: Guidance for protocols of clinical trials. BMJ. 2013;346:e7586

		Reporting Item	Page Number
**Administrative information**			
Title	#1	Descriptive title identifying the study design, population, interventions, and, if applicable, trial acronym	1
Trial registration	#2a	Trial identifier and registry name. If not yet registered,	1

For peer review only

		name of intended registry	
Trial registration: data set	#2b	All items from the World Health Organization Trial Registration Data Set	2
Protocol version	#3	Date and version identifier	2
Funding	#4	Sources and types of financial, material, and other support	10
Roles and responsibilities: contributorship	#5a	Names, affiliations, and roles of protocol contributors	10
Roles and responsibilities: sponsor contact information	#5b	Name and contact information for the trial sponsor	10
Roles and responsibilities: sponsor and funder	#5c	Role of study sponsor and funders, if any, in study design; collection, management, analysis, and interpretation of data; writing of the report; and the decision to submit the report for publication, including whether they will have ultimate authority over any of these activities	10
Roles and responsibilities: committees	#5d	Composition, roles, and responsibilities of the coordinating centre, steering committee, endpoint adjudication committee, data management team, and other individuals or groups overseeing the trial, if applicable (see Item 21a for data monitoring committee)	10

Introduction

For peer review only

Background and rationale	#6a	Description of research question and justification for undertaking the trial, including summary of relevant studies (published and unpublished) examining benefits and harms for each intervention	2-3
Background and rationale: choice of comparators	#6b	Explanation for choice of comparators	2-3
Objectives	#7	Specific objectives or hypotheses	1
Trial design	#8	Description of trial design including type of trial (eg, parallel group, crossover, factorial, single group), allocation ratio, and framework (eg, superiority, equivalence, non-inferiority, exploratory)	3
**Methods: Participants, interventions, and outcomes**			
Study setting	#9	Description of study settings (eg, community clinic, academic hospital) and list of countries where data will be collected. Reference to where list of study sites can be obtained	3
Eligibility criteria	#10	Inclusion and exclusion criteria for participants. If applicable, eligibility criteria for study centres and individuals who will perform the interventions (eg,	4

For peer review only

		surgeons, psychotherapists)	
Interventions: description	#11a	Interventions for each group with sufficient detail to allow replication, including how and when they will be administered	3
Interventions: modifications	#11b	Criteria for discontinuing or modifying allocated interventions for a given trial participant (eg, drug dose change in response to harms, participant request, or improving / worsening disease)	n/a
Interventions: adherance	#11c	Strategies to improve adherence to intervention protocols, and any procedures for monitoring adherence (eg, drug tablet return; laboratory tests)	3
Interventions: concomitant care	#11d	Relevant concomitant care and interventions that are permitted or prohibited during the trial	3
Outcomes	#12	Primary, secondary, and other outcomes, including the specific measurement variable (eg, systolic blood pressure), analysis metric (eg, change from baseline, final value, time to event), method of aggregation (eg, median, proportion), and time point for each outcome. Explanation of the clinical relevance of chosen efficacy and harm outcomes is strongly recommended	5-6
Participant timeline	#13	Time schedule of enrolment, interventions (including any run-ins and washouts), assessments, and visits for participants. A schematic diagram is highly recommended (see Figure)	10

For peer review only

Sample size	#14	Estimated number of participants needed to achieve study objectives and how it was determined, including clinical and statistical assumptions supporting any sample size calculations	5
Recruitment	#15	Strategies for achieving adequate participant enrolment to reach target sample size	3
**Methods: Assignment of interventions (for controlled trials)**			
Allocation: sequence generation	#16a	Method of generating the allocation sequence (eg, computer-generated random numbers), and list of any factors for stratification. To reduce predictability of a random sequence, details of any planned restriction (eg, blocking) should be provided in a separate document that is unavailable to those who enrol participants or assign interventions	3
Allocation concealment mechanism	#16b	Mechanism of implementing the allocation sequence (eg, central telephone; sequentially numbered, opaque, sealed envelopes), describing any steps to conceal the sequence until interventions are assigned	5
Allocation: implementation	#16c	Who will generate the allocation sequence, who will enrol participants, and who will assign participants to interventions	5

For peer review only

Blinding (masking)	#17a	Who will be blinded after assignment to interventions (eg, trial participants, care providers, outcome assessors, data analysts), and how	5
Blinding (masking): emergency unblinding	#17b	If blinded, circumstances under which unblinding is permissible, and procedure for revealing a participant's allocated intervention during the trial	5
**Methods: Data collection, management, and analysis**			
Data collection plan	#18a	Plans for assessment and collection of outcome, baseline, and other trial data, including any related processes to promote data quality (eg, duplicate measurements, training of assessors) and a description of study instruments (eg, questionnaires, laboratory tests) along with their reliability and validity, if known. Reference to where data collection forms can be found, if not in the protocol	6-7
Data collection plan: retention	#18b	Plans to promote participant retention and complete follow-up, including list of any outcome data to be collected for participants who discontinue or deviate from intervention protocols	6-7
Data management	#19	Plans for data entry, coding, security, and storage, including any related processes to promote data quality (eg, double data entry; range checks for data values).	6-7

For peer review only

		Reference to where details of data management procedures can be found, if not in the protocol	
Statistics: outcomes	#20a	Statistical methods for analysing primary and secondary outcomes. Reference to where other details of the statistical analysis plan can be found, if not in the protocol	6-7
Statistics: additional analyses	#20b	Methods for any additional analyses (eg, subgroup and adjusted analyses)	6-7
Statistics: analysis population and missing data	#20c	Definition of analysis population relating to protocol non-adherence (eg, as randomised analysis), and any statistical methods to handle missing data (eg, multiple imputation)	6-7
**Methods: Monitoring**			
Data monitoring: formal committee	#21a	Composition of data monitoring committee (DMC); summary of its role and reporting structure; statement of whether it is independent from the sponsor and competing interests; and reference to where further details about its charter can be found, if not in the protocol. Alternatively, an explanation of why a DMC is not needed	6
Data monitoring: interim analysis	#21b	Description of any interim analyses and stopping guidelines, including who will have access to these interim results and make the final decision to terminate the trial	6
Harms	#22	Plans for collecting, assessing, reporting, and managing solicited and spontaneously reported adverse events and other unintended effects of trial interventions or trial	6

For peer review only

		conduct	
Auditing	#23	Frequency and procedures for auditing trial conduct, if any, and whether the process will be independent from investigators and the sponsor	6
**Ethics and dissemination**			
Research ethics approval	#24	Plans for seeking research ethics committee / institutional review board (REC / IRB) approval	1
Protocol amendments	#25	Plans for communicating important protocol modifications (eg, changes to eligibility criteria, outcomes, analyses) to relevant parties (eg, investigators, REC / IRBs, trial participants, trial registries, journals, regulators)	1
Consent or assent	#26a	Who will obtain informed consent or assent from potential trial participants or authorised surrogates, and how (see Item 32)	1
Consent or assent: ancillary studies	#26b	Additional consent provisions for collection and use of participant data and biological specimens in ancillary studies, if applicable	n/a
Confidentiality	#27	How personal information about potential and enrolled participants will be collected, shared, and maintained in order to protect confidentiality before, during, and after the trial	9-10
Declaration of	#28	Financial and other competing interests for principal	10

For peer review only

interests		investigators for the overall trial and each study site	
Data access	#29	Statement of who will have access to the final trial dataset, and disclosure of contractual agreements that limit such access for investigators	10
Ancillary and post trial care	#30	Provisions, if any, for ancillary and post-trial care, and for compensation to those who suffer harm from trial participation	n/a
Dissemination policy: trial results	#31a	Plans for investigators and sponsor to communicate trial results to participants, healthcare professionals, the public, and other relevant groups (eg, via publication, reporting in results databases, or other data sharing arrangements), including any publication restrictions	10
Dissemination policy: authorship	#31b	Authorship eligibility guidelines and any intended use of professional writers	10
Dissemination policy: reproducible research	#31c	Plans, if any, for granting public access to the full protocol, participant-level dataset, and statistical code	10
**Appendices**			
Informed consent materials	#32	Model consent form and other related documentation given to participants and authorised surrogates	n/a
Biological specimens	#33	Plans for collection, laboratory evaluation, and storage of biological specimens for genetic or molecular analysis in the current trial and for future use in ancillary studies, if applicable	n/a

For peer review only

None The SPIRIT Explanation and Elaboration paper is distributed under the terms of the Creative Commons Attribution License CC-BY-NC. This checklist can be completed online using https://www.goodreports.org/, a tool made by the EQUATOR Network in collaboration with Penelope.ai

For peer review only
